# A genome annotation-driven approach to cloning the human ORFeome

**DOI:** 10.1186/gb-2004-5-10-r84

**Published:** 2004-09-30

**Authors:** John E Collins, Charmain L Wright, Carol A Edwards, Matthew P Davis, James A Grinham, Charlotte G Cole, Melanie E Goward, Begoña Aguado, Meera Mallya, Younes Mokrab, Elizabeth J Huckle, David M Beare, Ian Dunham

**Affiliations:** 1 The Wellcome Trust Sanger Institute, Wellcome Trust Genome Campus, Hinxton, Cambridge, CB10 1SA, UK; 2 MRC Rosalind Franklin Centre for Genomics Research (formerly MRC UK Human Genome Mapping Resource Centre), Hinxton, Cambridge, CB10 1SB, UK; 3 Current address: Department of Anatomy, University of Cambridge, Downing Street, Cambridge, CB2 3DY, UK; 4 Current address: Centro Nacional de Biotecnología (CNB), Campus de la Universidad Autónoma de Madrid, Cantoblanco, 28049 Madrid, Spain; 5 Current address: Cambridge Institute for Medical Research, Wellcome Trust/MRC Building, Addenbrooke's Hospital, Hills Road, Cambridge, CB2 2XY, UK; 6 Current address: Department of Biochemistry, Sanger Building, University of Cambridge, 80 Tennis Court Road, Cambridge, CB2 1GA, UK

## Abstract

We have developed a systematic approach to generating cDNA clones containing full-length open reading frames (ORFs), exploiting knowledge of gene structure from genomic sequence. Each ORF was amplified by PCR from a pool of primary cDNAs, cloned and confirmed by sequencing. We obtained clones representing 70% of genes on human chromosome 22, whereas searching available cDNA clone collections found at best 48% from a single collection and 60% for all collections combined.

## Abstract 2

Using a new systematic approach to generating cDNA clones containing full-length open reading frames, clones representing 70% of genes on human chromosome 22 were obtained.

## Background

Many methods for high-throughput, experimental elucidation of gene function (functional genomics) depend on the availability of full-length cDNA clone collections [1]. These clones provide access to the protein-coding open reading frames (ORFs) and facilitate expression of large numbers of proteins in the native form or as fusion proteins. The value of ORF-containing full-length cDNA clone collections (ORF clones) has now been amply demonstrated by studies in model organisms, in particular in the area of protein interaction mapping using methods based on yeast two-hybrid or mass spectrometry [2-8].

Extension of functional genomic approaches to mammalian genomes requires development of adequate ORF clone collections. Several projects based on complete sequencing of clones isolated from cDNA libraries are in place to generate these collections for mouse [9] and human [10-13]. Additional efforts have also focused on subsequent manipulation and exploitation of the full-length clones using versatile recombinational cloning systems so that the ORFs are formatted for expression [14-16]. However, obtaining a complete set of human clones has been hampered by the inadequacies of cDNA libraries and uncertainty over the true number and identity of all protein-coding genes. Approaches based on cDNA libraries have two major limitations in mammals. The first is the difficulty in obtaining full-length cDNA clones and the complexities of alternate and partial splice forms. Hence, many clones have to be sampled to obtain a canonical full-length version of each cDNA. The second is that these projects inevitably reach a point of diminishing return when it is no longer financially viable to continue to sequence more clones from the same library or from different tissues in order to add small numbers of new full-length cDNAs to the collection. Therefore, it is pertinent to ask how complete the cDNA collections currently are, and whether they can be supplemented or replaced by other approaches in order to develop complete ORF clone sets.

There is still uncertainty over the exact number of human and mouse genes and hence over the completeness of the existing cDNA collections. Therefore, we have investigated a defined subset of genes, namely the full-length protein-coding genes defined in our current annotation of human chromosome 22 [17]. In this study, we have found that in the currently available major cDNA collections, a total of 60% of chromosome 22 protein-coding genes are represented by complete ORF clones, although no single collection contains more than 48% (Table 1). This leaves a sizeable fraction of the genes unavailable. Thus there are still considerable challenges to be faced in identifying and isolating full-length cDNAs and ORFs for functional analyses.

To extend the coverage of full-length ORF clones, we have developed an alternative method which exploits knowledge of gene structure based on genomic sequence. It involves the specific amplification of a targeted ORF plus short regions of the 5' and 3' untranslated regions from a mixed pool of cDNAs. Amplified fragments are cloned into a standard plasmid sequencing vector and their identity and integrity confirmed by DNA sequencing. The aim of the method is to provide cDNA clones containing confirmed full-length ORFs, which can later be manipulated into suitable vector systems such as Gateway (Invitrogen) or Creator (BD Biosciences) for functional genomics. We have applied this method to the same set of chromosome 22 protein-coding genes and have shown that we can obtain clones representing 70% of the targeted genes with a limited range of experimental conditions. We have also demonstrated a reasonable expectation that we can isolate clones for 83%.

## Results and discussion

### Analysis of full-length cDNA collections

We have previously described a gene annotation of chromosome 22 [17] and its characterization [18]. In this annotation, 546 genes were defined as protein-coding genes, 387 being full length and the remainder (159) being partial, mostly as a result of unconfirmed 5' ends, incomplete genomic sequence or partial gene duplication events. We subsequently identified and removed two full-length genes which we now consider to be antisense transcripts and have extended 13 genes to full length to give a total of 398 full-length protein-coding genes (see [19] for details of the chromosome 22 ORFs). In the other cases of partial annotations we have not been able to extend the annotation sufficiently to allow identification of a complete ORF suitable for cloning. Therefore, for the purposes of this paper, where the aim is to identify clones containing complete ORFs, we only consider genes annotated as full-length protein coding as targets because of the difficulty of defining success for the partial genes.

We first considered the completeness of available full-length human cDNA collections, by comparing the DNA sequences of available cDNA library clones with our targeted set of 398 ORFs. For this analysis we used cDNA sequences downloaded from the major collections in January 2004. The publicly available cDNA collections analyzed were those from the Mammalian Gene Collection (MGC) [11], the full-length long Japan collection (FLJ) [12], the German cDNA Consortium (DKFZ) [10] and the Kazusa cDNA project (KIAA) [13]. In addition, we analyzed a commercially available set of cDNAs from Invitrogen. We aligned each of our target chromosome 22 ORFs to the available cDNA sequences to assess whether clones representing the entirety or any part of each of the chromosome 22 ORFs existed in each collection (Table 1, and see Materials and methods). This analysis showed that 240 out of 398 ORFs (60%) were represented by a cDNA clone with more than 95% identity over the full length of the ORF in at least one of the collections. In addition, a further 25 ORFs were covered by cDNA clones with gapped matches. However, only 227 (57% of the total ORFs) of these clones maintain the correct reading frame at the amino acid level. Examining the matches from individual cDNA clone collection showed that 80% of the full-length matches were provided by the MGC. This probably reflects the selection process in this program whereby initial sequencing of the ends of cDNA clones was used to select the optimal clone for complete sequencing. The KIAA collection provided full-length matches at approximately the same rate as the MGC, given the number of sequences available (1.25% chromosome 22 full-length matches out of the total MGC collection compared with 1.38% for KIAA) and notably provided the five largest clones matched that maintained the complete ORF (sizes between 4,719 base-pairs (bp) and 3,516 bp), reflecting the emphasis on long clones in the KIAA program. The FLJ and DKFZ collections gave rates of 0.28% and 0.27% respectively, presumably because a smaller proportion of full-length clones were sequenced. Analysis of the chromosome 22 genes from these collections shows that length, but not GC content, of the ORF is a significant factor in cloning success for these collections (Mann Whitney test, *p *< 0.0007), that is, there is bias against longer ORF clones.

In summary, there is currently a 60% chance of obtaining a full-length cDNA clone from one of these collections, based on a sample of 1% of the human genome. The best single collection (MGC) provides 48% of the clones. This analysis of coverage, based on the subset of full-length protein-coding genes on chromosome 22, mimics the situation occurring in a positional cloning type strategy where one might want to obtain clones for a region identified by genetic mapping. However, it does not assess whether the collections are enriched or depleted for specific classes of gene by function, tissue distribution or level of expression. As chromosome 22 is particularly GC-rich, and compared to other human chromosomes the set of genes we have used for this assessment may be biased towards housekeeping genes with widespread or ubiquitous expression which are known to be enriched in GC-rich regions of the genome. Hence, results for specific classes of genes will differ. In any case, one can expect to obtain roughly half of the clones required from one of these collections. This is testimony to the considerable effort that has gone into constructing the resources, but is also frustrating, because other sources are required to make up the substantial remainder. To investigate whether other approaches could be used to address the completeness of cDNA clone resources, we developed an alternative method which is complimentary to cDNA library sequencing, and tested this approach on the same set of chromosome 22 ORFs.

### Strategy for assembling a chromosome 22 ORF clone collection

**Figure 1:**
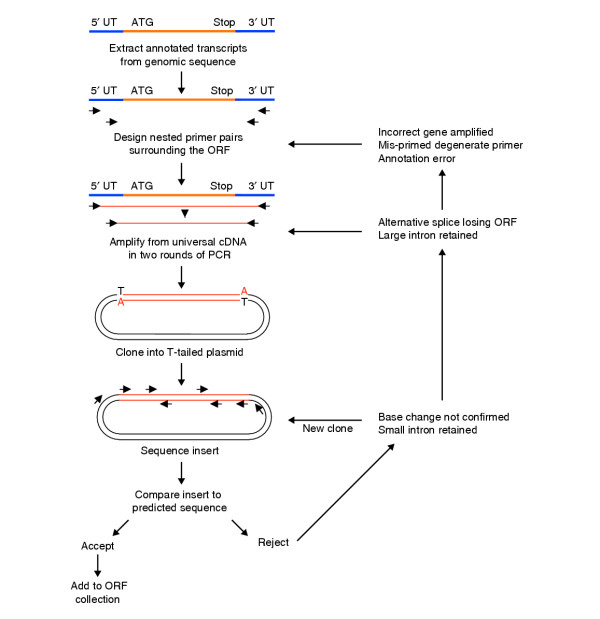
Summary of the ORF cloning method.

Previous efforts in human to obtain cDNA clones suitable for future functional genomics studies have started by isolating the longest possible cDNA clones [10-13]. In *Caenorhabditis elegans*, an alternative strategy has been developed that is directly tailored to clone ORFs defined by gene annotations from cDNA libraries into Gateway vectors ready for functional genomics [20]. The strategy we have developed (Figure 1) uses genome annotation to define the full-length ORFs of interest. We then aim to amplify the ORF bracketed by short sequences at either end from uncloned primary cDNA (rather than cDNA libraries) using reverse transcription (RT) PCR with modifications to allow efficient and high-throughput application. The overall aim is to obtain cDNA clones containing the defined set of ORFs more efficiently than by cDNA library screening and to access ORFs not present in existing cDNA library collections. This strategy enables a single protocol to be used for all genes, and therefore does not require the import of any previously existing cDNA clones which might be from multiple laboratories and in several vector systems. In addition, it avoids potential biases associated with cloned cDNA libraries by utilizing uncloned cDNA. We chose not to format the ORF directly for a specific recombinational cloning system because this might compromise our ability to isolate some ORFs by RT PCR. Furthermore ORFs cloned into a generic vector will be useful for those who do not want to use a specific vector format. ORFs in clones derived and verified by this method can be readily transferred into recombinational cloning systems by PCR with appropriately designed oligonucleotides.

For the 398 targets, a nested set of two pairs of PCR oligonucleotide primers surrounding each ORF and including a short region of the 5' and 3' untranslated regions was identified. As these primers were to be used to extract a fragment containing the ORF from an extremely complex cDNA template, design was not restricted to the sequences at the start and stop of the ORF. A highly processive, proof-reading thermostable DNA polymerase was use to amplify the ORF from a pool of cDNA derived from various tissues using two rounds of PCR. In 76% of cases amplification with KOD Hot Start polymerase was successful in generating a PCR product of expected size under one set of amplification conditions (see Additional data file 2). However, where the expected-sized PCR fragment was not obtained, we were often able to obtain a fragment by subsequent repeat of the procedure with slight modifications including increasing the annealing temperature, using *Pfu*-turbo DNA polymerase as an alternative enzyme for one or both rounds of PCR, or using a cDNA template from a single tissue rather than the pooled cDNA. Fragments of the correct size were cloned into a T-tailed plasmid and the inserts were verified by complete sequencing using vector primers and anticipated gene specific primers. Assembled sequence for each clone was then compared with the expected gene sequence. Clones were accepted as correct versions of the ORF if identical to the expected sequence or if they contained only base changes that were known to be single-nucleotide polymorphisms (SNPs) or resulted in silent codon changes. Clones were also accepted with an alternative splicing event that maintained the ORF. Clones were rejected (for this study) if they contained a nonsynonymous base change that could not be confirmed as a known SNP ('unconfirmed bases') or if they resulted from an alternative splice or partially processed mRNA that did not maintain the ORF. When a clone generated from a fragment of the correct size failed validation because of the presence of unconfirmed bases, or retention of a small intron, an alternative clone was picked and sequenced until a correct version was obtained. If alternative splicing or partial processing events gave unacceptable clones, a further round of reamplification was undertaken in order to obtain a correct fragment. Finally, if clone inserts were repeatedly unacceptable as a result of mispriming events, annotation error or amplification of a related gene, a new set of nested oligonucleotide primers were designed.

### Process error rate and SNPs

One possible concern with a strategy that involves reverse transcription and multiple rounds of PCR amplification followed by cloning of a single molecule is that the process will introduce base errors that alter the sequence of the final cloned ORF. Analysis of error rate here is complicated by the frequency of SNPs in humans and the fact that the starting cDNA template is a mix of cDNA from multiple human donors. We estimated the error rate from reverse transcription, PCR and the cloning process by sequencing 48 clones (covering 70,656 bases) containing the ORF of the NAGA gene. These were derived by our cloning protocol using cDNA from 10 lymphoblastoid cell lines as a template, as polymorphism would be easier to identify where each cDNA mix could only be one of two haplotypes. We categorized observed base changes as known SNPs if they were found to exist in dbSNP, in ESTs or in independently sequenced cDNA clones. Base changes were categorized as putative errors if no equivalent sequence could be identified. From this analysis we identified six putative base errors, giving an overall estimate of 0.085 errors per kilobase (kb), or one error per 7.8 clones assuming a mean ORF size of 1.5 kb.

### Chromosome 22 ORF clone collection

Applying the strategy outlined above to the 398 chromosome 22 ORFs, we were able to clone and confirm 278 (70%) of the targeted chromosome 22 ORFs (see Additional data file 1). Sequences of the valid ORF clones are available [19], and have been submitted to the EMBL database (accession numbers CR456339 to CR456616). Of these, 253 (91%) were derived from fragments generated with KOD polymerase. The remainder were generated using either an alternative polymerase (16; 6%) or a combination of polymerases (9; 3%) (see Additional data file 2). The universal cDNA pool was used for 249 (90%) of the clones, with 29 (10%) of clones derived from lower-complexity cDNA templates from single tissues. Of the accepted clones, 239 (86%) were the predicted splice form, with the remainder being an alternative splice which maintained the ORF; 183 (66%) clones matched the genomic DNA exactly. Of the 162 deviations from the genomic sequence (from 95 clones), 144 (89%) are previously identified SNPs either in dbSNP or dbEST, and 11 (7%) were not identified as known SNPs but did not alter the amino acid (see Additional data file 3). Seven changes were insertion/deletion events (see below). Of the 144 confirmed SNPs in a total of 372,916 bases (1 SNP every 2,590 bases), 81 were synonymous and 63 were nonsynonymous codon changes. Individual clones contained between one and eight SNPs (see Additional data file 3).

Insertions or deletions that retained the ORF were observed in five clones. None of these significantly altered the ORF, as four cases involved three bases while one involved 12 bases. We also observed a polymorphism in MSE55 which involved the insertion or deletion of six amino acid repeat units and exists in three different alleles. We amplified and sequenced genomic DNA fragments across this region from 152 chromosomes of European ancestry and found that all three alleles are common and in Hardy-Weinberg equilibrium. In this case the clone chosen for the ORF collection was the same allele as seen in the publicly available genomic sequence.

In three cases we obtained clones with insertion/deletion polymorphisms that altered the ORF but were supported by available chromosome 22 sequence. To determine whether to accept these clones as ORF cDNAs, we examined all three in more detail. The clone obtained for gene *APOL4 *contains a 2-bp insertion compared to the canonical genomic sequence annotation. This results in a frameshift that substantially extends the ORF from 127 amino acids to 348 amino acids. We designed a PCR reaction to directly interrogate the insertion/deletion and sequenced 144 chromosomes of European ancestry. Both alleles are common in this population, and are in Hardy-Weinberg equilibrium, with the 348-amino acid form being the minor allele at 46.5%. For bK216E10.6 we obtained an ORF clone with a 2-bp insertion compared to the genomic annotation, which results in an ORF that contains an extra 318 amino acids. Using the same strategy we sequenced 150 chromosomes and showed that the sequence producing the shorter peptide is the minor allele with a frequency of 20%, and the alleles are again in Hardy-Weinberg equilibrium. In this case we do not have an accepted clone, as the insertion increased the ORF length beyond the primer sequence. The third gene is *TXN2 *which shows a 2-bp insertion compared to the genomic sequence which is also found in an EST (AA586375), but has not been studied further. An insertion/deletion polymorphism that alters the ORF has previously been observed in *MICA *on chromosome 6 [21]. From these examples we concluded that insertion/deletion polymorphisms in ORFs that alter amino acid sequence may be relatively common, and can result in altered proteins. Complete ORF collections for outbred organisms like humans should ultimately address this issue and obtain examples of all common forms of the ORF.

In addition, we were able to amplify a PCR fragment which could be identified as originating from the correct gene for an additional 53 ORFs, but have not yet been able to obtain an acceptable clone because of the presence of unconfirmed bases, or problems with splice forms including partially processed transcripts. In most cases, only one or two amino acids are changed, which could make these clones usable under some circumstances, perhaps after site-directed mutagenesis. It is also possible that these are rarer SNPs that are not currently present in dbSNP. This suggests that by sequencing more examples we will be able to obtain clones for these ORFs in the near future. Thus the clone collection would cover 83% (331) of the targeted ORFs.

### Process failure

In total, we initiated the amplification and cloning process 538 times, excluding initial pilot trials. These 538 events break down as follows. For 180 (45%) targeted ORFs an acceptable clone was generated at the first attempt. Further rounds of clone-picking, reamplification or primer redesign generated a further 99 acceptable clones, 83 clones containing an unconfirmed base alteration, 54 clones containing an alternative splice which lost the ORF, 23 clones containing a rearrangement or erroneous amplification event, 19 clones with retained intron sequences, four clones containing unresolved sequencing problems and 36 clones which were not the expected gene. For 41 genes we were unable to amplify a suitable product or failed to clone the fragment. Hence the efficiency of the process in terms of the return of acceptable clones is approximately 52% (278/540).

**Figure 2:**
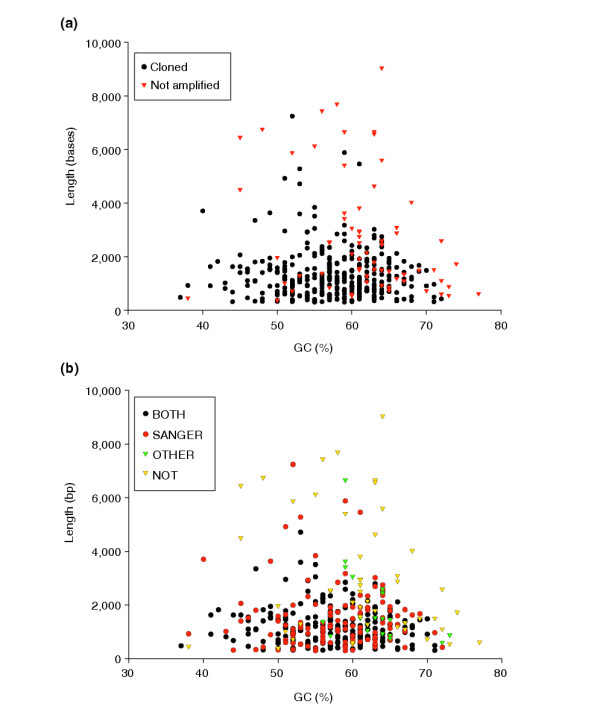
Sequence characteristics of cloned ORFs. **(a) **Plot of the distribution of the 398 chromosome 22 ORFs by GC content (%) and length (bases). Closed circles are the 331 ORFs that were isolated as acceptable clones (278) or as clones with the correct ORF but currently with a problem in the sequence (53). Dotted circles are the rest of the ORFs which were not amplifiable or clonable (67). **(b) **Overlap of chromosome 22 ORF clones isolated here with cDNA collections. Analysis of GC content and length for 398 chromosome 22 ORFs, split according to whether the gene has been isolated only by the strategy described here (SANGER, red circles), only in the cDNA collections (OTHER, green triangles), in both (BOTH, black circles), or not at all (NOT, yellow triangles).

A significant area of concern is where we were unable to generate a PCR product at all corresponding to the targeted gene. To find explanations for this type of failure, we examined both the sequence characteristics of the targeted ORF and elements of the experimental design. First we examined the crude differences between the classes of ORFs that we could and could not amplify. Figure 2a shows a plot of the distributions of these two classes by GC content and length of ORF. Both GC content and length are significant predictors of success/failure to amplify (Mann Whitney test *p *< 0.0001), although logistic regression indicates there is no significant interaction between them. This suggests that alternative amplification protocols using different polymerases or PCR additives might result in additional ORFs being obtained. However, we have tested three additional enzymes or mixes (*Pfu *Ultra (Stratagene), Phusion (Finnzymes) and Expand 20 kb+ PCR (Roche)) and additives including DMSO, glycerol and betaine so far without identifying a design that solves the problem.

Next, we explored whether it was possible to amplify any part of the failed target cDNAs from the universal mix. For 51 of the genes where we failed to amplify the expected fragment, we designed additional nested oligonucleotide primer pairs to amplify a short (100-274 bp) sequence across a splice junction. In 39 cases (74%) we amplified a fragment of the correct size and sequence under our standard nested PCR conditions, suggesting that template is present in the cDNA mix for these ORFs (data not shown). Therefore, in most cases it is possible to amplify part of the targeted ORF from the cDNA mix using this protocol, indicating that the level of target in the mix is not limiting in these cases. Given that we know we can amplify parts of many of the problematic genes, one variation that could improve access to larger ORFs in the future would be to amplify larger transcripts in pieces that can then be reassembled into a single clone using appropriate restriction enzyme digestion and ligation or PCR cloning methods.

We also examined whether successful amplification was biased towards genes expressed in many tissues. Su *et al*. [22] have generated microarray data indicating the distribution of expression for many human genes over 47 tissues. We downloaded these data [23] and were able to obtain tissue-distribution data for 206 of our 398 targeted genes. Codifying the diversity of tissues in which the genes were expressed as the proportion of positive tissues, and analyzing for the success or failure of amplification by logistic regression, indicated that the probability of amplifying a gene is not significantly affected by the diversity of its expression (data not shown).

We also examined diversity of expression by analyzing serial analysis of gene expression (SAGE) data derived from 242 *Nla*III SAGE libraries downloaded from the SAGEmap resource [24]. SAGE tags could be uniquely mapped to 315 of the 398 ORFs targeted. Using the number of SAGE libraries in which a SAGE tag for an ORF was found to represent the diversity of tissues in which the gene was expressed, no significant relationship was found with the probability of amplifying a gene (Mann Whitney test, *p *= 0.84). Furthermore, because the SAGE tag data also gives an indication of expression level, we examined whether the mean expression level found by SAGE (mean normalized tags per million SAGE reads) affected probability of expression and again found no significant relationship (Mann Whitney test, *p *= 0.79). Taken together these analyses indicate that the success of our amplification strategy is not significantly influenced by either the range of tissues in which a gene is expressed or the level of expression. Clearly there will be some genes expressed at low levels, at specific times or in specific tissues that will need special treatment, but these data suggest that these cases may be few.

### Comparison of the chromosome 22 ORF collection with other cDNA sources

**Figure 3:**
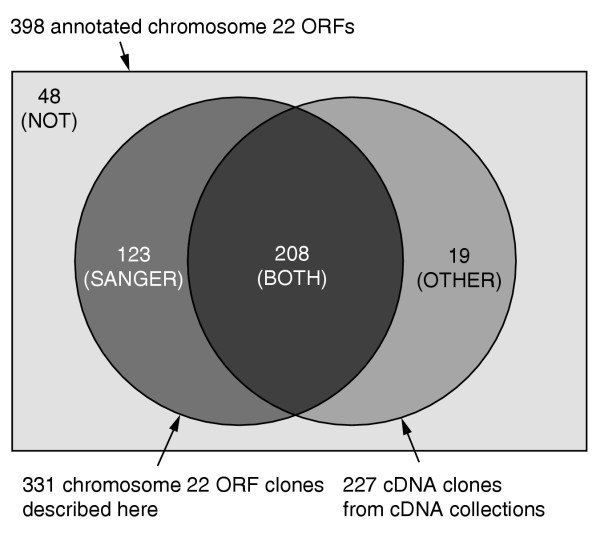
Schematic Venn diagram showing the relationships of the set of ORF clones isolated here compared with the full-length cDNA clones in current high-throughput clone collections (227 maintain the correct reading frame at the amino acid level from Table 1) for the 398 annotated full-length chromosome 22 ORFs. The four different classes of genes are labeled as in the text and Figure 2b.

Returning to the cDNA clone collections, of the 331 targeted genes for which we can obtain either an acceptable clone (278) or a clone of the correct ORF but currently with a problem in its sequence (53), 208 genes also have clones in the cDNA clone collections we analyzed; 123 genes only have clones in the new chromosome 22 ORF set described here. In addition, for 19 genes which are represented in the cDNA clone collections we were unable to isolate a clone (Figures 2b, 3). This means that 88% (350) of the full-length protein-coding genes on chromosome 22 have cDNA clones. This also suggests that achieving 88% coverage of the readily accessible human ORFeome should be possible with an approach that combines the existing cDNA collections with directed RT-PCR as implemented in this analysis. Of course, because the actual number of human genes is still unknown and a significant number of genes have only partial annotation, there is still an indeterminate number of genes for which there is insufficient annotation to attempt the current strategy.

We analyzed the four classes of genes (isolated by us and in the cDNA collections (BOTH), isolated only here (SANGER), isolated only by the cDNA collections (OTHER) and not isolated (NOT)) by GC content, length and diversity of expression as defined above for microarray data and SAGE using nonparametric analysis of variance (Figure 3, and Additional data file 5). ORF length was significantly higher (*p *< 0.001) for genes not isolated (NOT) as compared to those isolated by us (SANGER) or those isolated both by us and the cDNA collections (BOTH). This suggests, as expected, that longer ORFs are harder to amplify or clone. A significant influence (*p *< 0.05) was also found for higher GC content in the genes that were either not isolated (NOT) or found only in the cDNA collections (OTHER) compared with the SANGER or BOTH classes, reflecting the influence of GC content on the ability to amplify a cDNA target as discussed above. The only significant difference (*p *< 0.05) for diversity of expression was between genes cloned only by us (SANGER) and those present in both our set and the cDNA collections (BOTH), with less diversely expressed genes slightly enriched in the SANGER class. This result was seen only in the microarray data, although the effect was also present in the SAGE data at just below significance. This suggests that the method described here may be able to access less widely expressed genes than have been sampled by existing cDNA library sequencing, although the effect is small. Finally, analysis of the mean level of expression of the genes in the four classes based on the normalized SAGE tag count showed no significant difference, indicating that level of expression is not a significant factor for this set of genes.

## Conclusions

Even given a high-quality human genome sequence, we still face considerable challenges in identifying and isolating full-length cDNAs and ORFs in order to construct genome-wide clone sets for functional analyses. The method we have described here offers an alternative approach to obtaining full-length ORF clones compared with sequencing or amplifying from cDNA libraries. We have demonstrated that we can readily obtain clones for 70% of the full-length protein-coding genes on chromosome 22, increasing to 83% if we include the largely correct clones which have not passed the confirmation criteria. In addition, a small number of clones (19) that we could not obtain are present in the cDNA collections analyzed, and when these are included, the overall coverage of the known full-length protein-coding genes reaches 88%. While this represents a substantial gain over cDNA sequencing alone, it is clear that complete coverage may require further modification of the approach or additional strategies as well.

The quality control that is introduced by starting with annotated genes on the genomic sequence allows identification of SNPs and artifacts within the clones, and allows confirmation or rejection of each clone as it is generated. The checking process also provides verification of gene structures annotated from assembled ESTs, and in a few cases revealed errors. Our approach also has some advantages for scale-up to whole genomes. The starting point is a single PCR reaction using a universal template, which could be adapted to standard automation platforms. Subsequent steps, including ligation, transformation, clone picking, sequencing and sequence analysis, are all amenable to existing robotic approaches or automation. At present, the gel-purification step of the amplified PCR fragment might be difficult to automate. It is also likely that the final sign-off on the sequence alignment of clones will require human intervention in much the same way as finishing genomic sequences does. However, application to whole genomes demands a high-quality gene annotation to be available for the whole genome.

We have generated a set of quality-controlled ORFs surrounded by a short stretches of 5' and 3' untranslated sequence in a uniform vector. The ORF portions of these intermediary clones are currently being amplified and subcloned in frame into a mammalian expression vector which fuses the amino-terminal T7 phage major capsid protein to the amino or carboxy terminus of the protein. We have successfully performed subcellular localization studies using immunofluorescence microscopy with these clones. We are also transferring the ORFs into Gateway pDONR clones (Invitrogen) and subsequently using GFP fusion destination vectors for subcellular localization. The availability of the ORF in a generic vector provides flexibility in the future downstream formats in that the endogenous Kozak sequence and the translation start and stop are maintained, and without additional amino acids from recombination sites. Finally, it is worth noting that this approach could also be applied to amplifying and cloning the many alternatively spliced forms of genes, or ORFs from different individuals or haplotypes. The ability to access the many additional variants beyond the canonical ORFeome could prove a valuable tool for future studies.

## Materials and methods

### cDNA sequence sources and websites

cDNA sequences were downloaded from the websites of the following publicly available cDNA collections in January 2004. For the Mammalian Gene Collection (MGC [11,25], 15,454 sequences were downloaded on 16 January 2004 [26]. For the full-length long Japan collection (FLJ [12]), 25,696 sequence accession numbers were obtained on 16 January 2004 [27] and the sequences were downloaded from the EMBL sequence database. For the German cDNA Consortium (DKFZ [10]) we identified 9,271 sequence accessions on 16 January 2004 [28] and sequences were downloaded from the EMBL database. For the Kazusa cDNA project (KIAA [13,29]), 2,037 sequence accession numbers were obtained on 26 January 2004 [29], and sequences were downloaded from the EMBL database, although two cDNA sequences were missing (KIAA0013 and KIAA0302). In addition, we downloaded 4,361 of the commercially available Invitrogen cDNAs on 8 December 2003 [30] (file datestamp 20 October 2003).

### Amplification and cloning of ORFs

Chromosome 22 gene annotations containing full-length ORFs, as defined in Collins *et al. *[17], but not including the genes described as possible antisense, and 13 genes subsequently completed, provided 398 complete chromosome 22 gene sequences. Nested sets of two pairs of PCR primers surrounding each ORF were designed using Primer3 (Steve Rozen, Helen J. Skaletsky (1996, 1997), Primer3, Code available at [31]) and Perl (version 5.004) scripts to automate the process (see Additional data file 4 for primer pairs designed). Fragments were amplified with the outer primer pair from either 0.1 ng of a pool of cDNAs from 37 tissues (Human Universal QUICK-Clone cDNA, Clontech), or cDNA from a single tissue (cervix, liver, brain, testis, fetal liver or fetal brain obtained as RNA from Stratagene or QUICK-Clone cDNA from Clontech), or cDNA from lymphoblastoid cell lines (European Collection of Cell Cultures, Porton Down, UK HRC collection, cell lines lines C0043, C0092, CO118, C0127, C0139, C0143, C0155, C0167, C0179, C0259, C0573). For the lymphoblastoid cells lines, total RNA was extracted from tissue culture cells with TRIzol reagent (GibcoBRL/Invitrogen). Total RNA was reverse transcribed into cDNA with Superscript II (Invitrogen) according to the manufacturer's instructions. The first-round amplification protocol used KOD Hot Start DNA polymerase (Novagen), *Pfu*-turbo Hotstart DNA polymerase (Stratagene) or *Pfu *DNA polymerase (Stratagene) using the manufacturers' recommended cycling profiles for 30 or 35 cycles in a 25 μl reaction. Fragments were then diluted 1 in 50 with sterile water and 5 μl used as template for a second 25 μl amplification using the inner primer pair (see Additional data file 2 for variant amplification conditions). Additional enzymes including *Pfu *Ultra (Stratagene), Phusion (Finnzymes) and Expand 20 kb+ PCR (Roche) were trialed according to the manufacturers' recommendations. Fragments of the expected size were gel-purified, extracted with QIAquick spin columns (Qiagen), 3'-tailed with an adenosine residue using Amplitaq polymerase (Perkin Elmer) and subcloned using the pGEM-T Easy Vector System (Promega). Sequencing template was prepared either by plasmid miniprep or, in the majority of cases, by amplifying clone inserts with vector primers and cleaning the amplified fragment with either QIAquick Gel Extraction Kit or Shrimp alkaline phosphatase (1 unit, Amersham) and ExonucleaseI (1 unit, Amersham) (see below). Sequencing was performed with BigDye terminator v3 Cycle Sequencing Kits (Applied Biosystems) using vector primers, the inner nested primer pair and pairs of primers designed at 600-base intervals along the predicted gene sequence. Sequence was assembled using the contig assembly program CAP3 [32], aligned against the predicted transcript sequence and checked manually.

### Sequence comparison and analysis

The 398 annotated ORF sequences were matched by blastn (version 2.0 MP-WashU, 10 April 2004 [33]) to cDNA collection databases MGC, FLJ, DKFZ, KIAA and Invitrogen. MSPcrunch [34] was used to parse blastn output and exclude matches with lower than 95% identity. ORFs were extracted from each of the matching cDNA sequences using the EMBOSS program getorf [35] and compared to the annotated ORFs using cross_match (P. Green, unpublished work). The GC content of the ORFs was calculated using the EMBOSS program geecee [35] and Perl scripts (version 5.004) were written to analyze and summarize data.

Microarray data indicating the distribution of expression for many human genes over 47 tissues using the Affymetrix human U95A array [22] was downloaded [23]. Tissue distribution data for 206 genes was obtained and a gene was called as expressed in a tissue sample if the average difference was > 200 [22]. The tissue expression diversity of a gene was defined as the proportion of positive tissues. Where replicate experiments existed, the highest tissue-expression diversity value was used.

For SAGE data, the 398 target ORF sequences were matched by blastn [33] against Unigene (Homo sapiens, 12 May 2004 Build 170 [36]) and the best (highest identity greater than 99%) full-length matching UniGene cluster was assigned to each ORF. SAGEmap data [24] was downloaded [37] together with a file of tag frequencies [38].

A Perl program was then used to search these files for SAGE tags mapping to each UniGene cluster and the tag counts for each *Homo sapiens Nla*III library (GPL4) were determined. Tag counts were normalized to tags per million for each library, and then averaged to give a mean expression level. Diversity of expression was defined as the number of libraries in which a tag occurred.

### Amplification and sequencing of genomic DNA for insertion/deletion analysis

Fifty nanograms of genomic DNA from 78 unrelated individuals (ECACC Human Random Control Panel) was amplified in 15 μl reactions containing: 6.7 mM MgCl_2_, 67 mM Tris-HCl, 16.7 mM (NH_4_)_2_SO_4 _pH 8.8, 170 μg/ml BSA, 10 mM 2-mercaptoethanol, 500 μM each dATP, dCTP, dGTP, dTTP, 0.04 units/μl Amplitaq, 0.75 μM each primer, 10.13% sucrose, 0.0029% Cresol Red (sodium salt). Reactions were cycled in an MJ thermocycler at 94°C for 5 min, followed by 35 cycles of 30 seconds at 94°C; 30 sec at 65-66°C; 30 sec at 72°C, followed by a final 72°C for 5 min. PCR reactions were treated with 1 unit of shrimp alkaline phosphatase and 1 unit of exonuclease I in reaction buffer supplied by the manufacturer (USB, 10 × buffer - 200 mM Tris-HCl pH 8, 100 mM MgCl_2_) for each 10 μl PCR reaction. Reactions were heated at 37°C for 30 min followed by 80°C for 15 min to inactivate the enzymes. PCR products were then sequenced from both ends using the primers used from the amplification step and BigDye terminator v3 Cycle Sequencing Kits (Applied Biosystems). Sequences were analyzed using GAP4 [39].

## Additional data files

The following additional data files are available with the online version of this article. Additional data file 1 lists the 278 successfully cloned ORFs (see also [19]); Additional data file 2 lists enzymes and templates used to amplify ORFs; Additional data file 3 lists the sequence variation between ORF clone and genomic sequence; Additional data file 4 lists the nested oligonucleotide primers designed for the 398 targeted genes; Additional data file 5 contains the results of nonparametric ANOVA (Kruskal-Wallis Test) for chromosome 22 genes isolated as cDNA by the method described here only (SANGER), found in the cDNA collections only (OTHER), isolated by both ourselves and the cDNA collections (BOTH) or not isolated (NOT). Mean rank differences and *p*-values are given after Dunn's multiple comparisons test. Additional data file 6 contains a list of the 278 cloned ORFs and Additional data file 7 contains a list of the 398 target ORFs; both files are also available at [19].

## Supplementary Material

Additional data file 1 The 278 successfully cloned ORFs Click here for additional data file

Additional data file 2 Enzymes and templates used to amplify ORFs Click here for additional data file

Additional data file 3 The sequence variation between ORF clone and genomic sequence Click here for additional data file

Additional data file 4 The nested oligonucleotide primers designed for the 398 targeted genes Click here for additional data file

Additional data file 5 The results of nonparametric ANOVA (Kruskal-Wallis Test) for chromosome 22 genes isolated as cDNA by the method described here only (SANGER), found in the cDNA collections only (OTHER), isolated by both ourselves and the cDNA collections (BOTH) or not isolated (NOT) Click here for additional data file

Additional data file 6 A list of the 278 cloned ORFs Click here for additional data file

Additional data file 7 A list of the 398 target ORFs Click here for additional data file

## Figures and Tables

**Table 1:** Analysis of genome-wide collections

cDNA collection	Total cDNAs available	Matches to 398 chromosome 22 ORFs at more than 95% identity*				
		
		Exact match	Gapped match	5' end match	3' end match	Internal match
MGC	15,454	193	14	21	23	17
FLJ	25,696	72	24	25	75	25
DKFZ	9,271	25	10	3	49	16
KIAA	2,035	28	1	1	18	13
Invitrogen	4,361	16	0	61	1	17
Combined	56,817	240^†^	25	27	39	14

*For definitions of match types see Materials and methods. Values are not significantly altered by raising the identity required to >99%. ^†^Only 227 (57% of the total ORFs) of these clones maintain the correct reading frame at the amino acid level.
