# Supplementary material for: A genome annotation-driven approach to cloning the human ORFeome
Source: Genome Biol. 2004 Sep 30;5(10):R84. doi: 10.1186/gb-2004-5-10-r84 (PMC545604; doi:10.1186/gb-2004-5-10-r84)
Supplement: Additional data file 1 — The 278 successfully cloned ORFs [file gb-2004-5-10-r84-s1.doc]

Supplementary Table 1. The 278 successfully cloned ORFs

| **Locus** | **Target CDS** | **Clone Name** | **Accession No.** |
| --- | --- | --- | --- |
| Em:AC000068.C22.3 | Em:AC000068.C22.3 | pGEM.Em:AC000068.3 | CR456339 |
| CYP2D6 | Em:AC002073.C22.2 | pGEM.Em:AC002073.2.V3 | CR456340 |
| Em:AC002378.C22.1 | Em:AC002378.C22.1 | pGEM.Em:AC002378.1 | CR456341 |
| Em:AC002472.C22.7 | Em:AC002472.C22.7 | pGEM.Em:AC002472.7 | CR456342 |
| Em:AC002472.C22.8 | Em:AC002472.C22.8 | pGEM.Em:AC002472.8 | CR456343 |
| Em:AC004471.C22.1 | Em:AC004471.C22.1 | pGEM.Em:AC004471.1.V2 | CR456344 |
| Em:AC004832.C22.1 | Em:AC004832.C22.1 | pGEM.Em:AC004832.1.V2 | CR456345 |
| Em:AC004832.C22.5 | Em:AC004832.C22.5 | pGEM.Em:AC004832.5.V5 | CR456346 |
| Em:AC004997.C22.11 | Em:AC004997.C22.11 | pGEM.Em:AC004997.11 | CR456347 |
| Em:AC005003.C22.4 | Em:AC005003.C22.4 | pGEM.Em:AC005003.4 | CR456348 |
| Em:AC005005.C22.6 | Em:AC005005.C22.6 | pGEM.Em:AC005005.6 | CR456349 |
| Em:AC005006.C22.2 | Em:AC005006.C22.2 | pGEM.Em:AC005006.2 | CR456350 |
| Em:AC005006.C22.4 | Em:AC005006.C22.4 | pGEM.Em:AC005006.4 | CR456351 |
| Em:AC005500.C22.4 | Em:AC005500.C22.4 | pGEM.Em:AC005500.4.V2 | CR456352 |
| Em:AC005529.C22.5 | Em:AC005529.C22.5 | pGEM.Em:AC005529.5.V2 | CR456353 |
| Em:AC006547.C22.2 | Em:AC006547.C22.2 | pGEM.Em:AC006547.2 | CR456354 |
| Em:AC006547.C22.3 | Em:AC006547.C22.3 | pGEM.Em:AC006547.3.V2 | CR456355 |
| Em:AC006547.C22.4 | Em:AC006547.C22.4 | pGEM.Em:AC006547.4.V3 | CR456356 |
| Em:AC006547.C22.5 | Em:AC006547.C22.5 | pGEM.Em:AC006547.5 | CR456357 |
| Em:AC006547.C22.7 | Em:AC006547.C22.7 | pGEM.Em:AC006547.7 | CR456358 |
| Em:AC007050.C22.6 | Em:AC007050.C22.6 | pGEM.Em:AC007050.6 | CR456359 |
| Em:AC007663.C22.1 | Em:AC007663.C22.1 | pGEM.Em:AC007663.1 | CR456360 |
| Em:AC007663.C22.3 | Em:AC007663.C22.3 | pGEM.Em:AC007663.3 | CR456361 |
| Em:AC008101.C22.3 | Em:AC008101.C22.3 | pGEM.Em:AC008101.3 | CR456362 |
| Em:AC008101.C22.5 | Em:AC008101.C22.5 | pGEM.Em:AC008101.5 | CR456363 |
| Em:AC016026.C22.2 | Em:AC016026.C22.2 | pGEM.Em:AC016026.2 | CR456364 |
| ACO2 | dJ347H13.C22.1 | pGEM.ACO2 | CR456365 |
| ACR | Em:AC000036.C22.1 | pGEM.ACR.V3 | CR456366 |
| ADORA2A | Em:AP000355.C22.1 | pGEM.ADORA2A.V2 | CR456367 |
| ADSL | dJ1042K10.C22.1.2 | pGEM.ADSL | CR456368 |
| ALG12 | fF1A6.C22.1 | pGEM.ALG12.V3 | CR456369 |
| Em:AP000346.C22.6 | Em:AP000346.C22.6c | pGEM.Em:AP000346.6 | CR456370 |
| Em:AP000348.C22.3 | Em:AP000348.C22.3 | pGEM.Em:AP000348.3.V2 | CR456371 |
| Em:AP000350.C22.1 | Em:AP000350.C22.1 | pGEM.Em:AP000350.1 | CR456372 |
| Em:AP000350.C22.2 | Em:AP000350.C22.2 | pGEM.Em:AP000350.2.V2 | CR456373 |
| Em:AP000351.C22.3 | Em:AP000351.C22.3 | pGEM.Em:AP000351.3 | CR456374 |
| Em:AP000355.C22.2 | Em:AP000355.C22.2 | pGEM.Em:AP000355.2 | CR456375 |
| Em:AP000553.C22.3 | Em:AP000553.C22.3 | pGEM.Em:AP000553.3 | CR456376 |
| Em:AP000557.C22.1 | Em:AP000557.C22.1 | pGEM.Em:AP000557.1.V3 | CR456377 |
| Em:AP000557.C22.3 | Em:AP000557.C22.3 | pGEM.Em:AP000557.3.V6 | CR456378 |
| APOL3 | bK212A2.C22.1b | pGEM.APOL3 | CR456379 |
| APOL4 | bK212A2.C22.5 | pGEM.APOL4.V2 | CR456380 |
| APOL6 | fF62D4.C22.2 | pGEM.APOL6 | CR456381 |
| ARFGAP1 | dJ437M21.C22.1 | pGEM.ARFGAP1 | CR456382 |
| ARSA | Em:U62317.C22.8 | pGEM.ARSA.V2 | CR456383 |
| ATF4 | dJ1104E15.C22.2 | pGEM.ATF4.V4 | CR456384 |
| ATP6E | Em:AC006285.C22.6 | pGEM.ATP6E.V2 | CR456385 |
| bA247I13.C22.2 | bA247I13.C22.2 | pGEM.bA247I13.2 | CR456386 |
| bA494O16.C22.1 | bA494O16.C22.1 | pGEM.bA494O16.1.V3 | CR456387 |
| bA9F11.C22.1 | bA9F11.C22.1 | pGEM.bA9F11.1 | CR456388 |
| BID | Em:AC006285.C22.1 | pGEM.BID | CR456389 |
| BIK | bK1191B2.C22.2 | pGEM.BIK.V2 | CR456390 |
| bK1048E9.C22.3 | bK1048E9.C22.3 | pGEM.bK1048E9.3.V2 | CR456391 |
| bK1048E9.C22.4 | bK1048E9.C22.4 | pGEM.bK1048E9.4 | CR456392 |
| bK150C2.1 | bK150C2.C22.1 | pGEM.bK150C2.1.V3 | CR456393 |
| bK150C2.3 | bK150C2.C22.3 | pGEM.bK150C2.3 | CR456394 |
| bK150C2.4 | bK150C2.C22.4 | pGEM.bK150C2.4 | CR456395 |
| bK150C2.9 | bK150C2.C22.9 | pGEM.bK150C2.9 | CR456396 |
| bK175E3.C22.6 | bK175E3.C22.6 | pGEM.bK175E3.6.V4 | CR456397 |
| bK223H9.2 | bK223H9.C22.2 | pGEM.bK223H9.2 | CR456398 |
| bK250D10.2 | bK250D10.C22.2 | pGEM.bK250D10.2 | CR456399 |
| bK250D10.C22.6 | bK250D10.C22.6 | pGEM.bK250D10.6 | CR456400 |
| bK250D10.C22.8 | bK250D10.C22.8 | pGEM.bK250D10.8 | CR456401 |
| bK268H5.C22.1 | bK268H5.C22.1 | pGEM.bK268H5.1 | CR456402 |
| bK268H5.C22.4 | bK268H5.C22.4 | pGEM.bK268H5.4 | CR456403 |
| bK29F11.C22.1 | bK29F11.C22.1 | pGEM.bK29F11.1 | CR456404 |
| bK747E2.C22.1 | bK747E2.C22.1 | pGEM.bK747E2.1 | CR456405 |
| bK963H5.C22.1 | Em:AC003072.C22.1 | pGEM.bK963H5.1 | CR456406 |
| bK984G1.1 | bK984G1.C22.1 | pGEM.bK984G1.1 | CR456407 |
| BRD1 | dJ522J7.C22.2 | pGEM.BRD1 | CR456408 |
| BZRP | dJ526I14.C22.1b | pGEM.BZRP.V2 | CR456409 |
| C22orf2 | dJ508I15.C22.1 | pGEM.C22orf2 | CR456410 |
| C22orf3 | bK984G1.C22.2 | pGEM.C22orf3 | CR456411 |
| C22orf4 | fF64F4.C22.1 | pGEM.C22orf4 | CR456412 |
| C22orf5 | dJ5O6.C22.2 | pGEM.C22orf5 | CR456413 |
| CACNG2 | dJ293L6.C22.1 | pGEM.CACNG2 | CR456414 |
| cB5E3.C22.2 | cB5E3.C22.2 | pGEM.cB5E3.2.V4 | CR456415 |
| cE81G9.C22.2 | cE81G9.C22.2 | pGEM.cE81G9.2 | CR456416 |
| CECR1 | Em:AC005300.C22.1 | pGEM.CECR1 | CR456417 |
| CHEK2 | bA444G7.C22.1 | pGEM.CHEK2 | CR456418 |
| CHKL | Em:U62317.C22.6 | pGEM.CHKL.V2 | CR456419 |
| CLDN5 | Em:AC000088.C22.1 | pGEM.CLDN5.V3 | CR456420 |
| cN5H6.C22.4 | cN5H6.C22.4 | pGEM.cN5H6.4 | CR456421 |
| COMT | Em:AC000080.C22.1 | pGEM.COMT | CR456422 |
| CRKL | Em:AC002470.C22.1 | pGEM.CRKL | CR456423 |
| CRYBA4 | bK445C9.C22.2 | pGEM.CRYBA4.V3 | CR456424 |
| CRYBB1 | bK445C9.C22.1 | pGEM.CRYBB1 | CR456425 |
| CRYBB2 | bK221G9.C22.3 | pGEM.CRYBB2 | CR456426 |
| CRYBB3 | bK221G9.C22.2 | pGEM.CRYBB3 | CR456427 |
| CSF2RB | bK833B7.C22.2 | pGEM.CSF2RB.V2 | CR456428 |
| CSNK1E | dJ5O6.C22.1 | pGEM.CSNK1E | CR456429 |
| CYP2D6 | Em:M33388.CDS | pGEM.CYP2D6.V2 | CR456430 |
| DDT | Em:AP000351.C22.2 | pGEM.DDT | CR456431 |
| DDX17 | dJ434P1.C22.1 | pGEM.DDX17 | CR456432 |
| DGCR2 | Em:AC000095.C22.1 | pGEM.DGCR2.V2 | CR456433 |
| DGCR6 | Em:AC008103.C22.4 | pGEM.DGCR6 | CR456434 |
| DIA1 | dJ222E13.C22.4 | pGEM.DIA1 | CR456435 |
| dJ1014D13.C22.1 | dJ1014D13.C22.1 | pGEM.dJ1014D13.1 | CR456436 |
| dJ1014D13.C22.2 | dJ1014D13.C22.2 | pGEM.dJ1014D13.2 | CR456437 |
| dJ102D24.C22.2 | dJ102D24.C22.2 | pGEM.dJ102D24.2 | CR456438 |
| dJ1033E15.C22.2 | dJ1033E15.C22.2 | pGEM.dJ1033E15.2 | CR456439 |
| dJ1039K5.C22.6 | dJ1039K5.C22.6 | pGEM.dJ1039K5.6 | CR456440 |
| dJ1042K10.2 | dJ1042K10.C22.2 | pGEM.dJ1042K10.2 | CR456441 |
| dJ1057D18.1 | dJ1057D18.C22.1 | pGEM.dJ1057D18.1 | CR456442 |
| dJ1104E15.C22.4 | dJ1104E15.C22.4 | pGEM.dJ1104E15.4 | CR456443 |
| dJ1119A7.C22.3 | dJ1119A7.C22.3 | pGEM.dJ1119A7.3 | CR456444 |
| dJ1163J1.C22.4 | dJ1163J1.C22.1 | pGEM.dJ1163J1.4.V3 | CR456445 |
| dJ1170K4.C22.2 | dJ1170K4.C22.2 | pGEM.dJ1170K4.2 | CR456446 |
| dJ1198O21.C22.1 | Em:AC004542.C22.2 | pGEM.dJ1198O21.1.V3 | CR456447 |
| dJ127B20.C22.3 | dJ127B20.C22.3 | pGEM.dJ127B20.3.V2 | CR456448 |
| dJ130H16.C22.1 | Em:AC004997.C22.1 | pGEM.dJ130H16.1.V3 | CR456449 |
| dJ149A16.6 | dJ149A16.C22.6 | pGEM.dJ149A16.6 | CR456450 |
| dJ151B14.C22.4 | dJ151B14.C22.4 | pGEM.dJ151B14.4.V4 | CR456451 |
| dJ172B20.C22.3 | dJ172B20.C22.3 | pGEM.dJ172B20.3 | CR456452 |
| dJ186O1.1 | dJ186O1.C22.1 | pGEM.dJ186O1.1.V4 | CR456453 |
| dJ186O1.2 | dJ186O1.C22.2 | pGEM.dJ186O1.2.V2 | CR456454 |
| dJ222E13.C22.1 | dJ222E13.C22.1 | pGEM.dJ222E13.1.V4 | CR456455 |
| dJ222E13.C22.3 | dJ222E13.C22.3a | pGEM.dJ222E13.3 | CR456456 |
| dJ347H13.2 | dJ347H13.C22.2 | pGEM.dJ347H13.2 | CR456457 |
| dJ347H13.4 | dJ347H13.C22.4 | pGEM.dJ347H13.4 | CR456458 |
| dJ347H13.5 | dJ347H13.C22.5 | pGEM.dJ347H13.5 | CR456459 |
| dJ355C18.1 | dJ355C18.C22.1 | pGEM.dJ355C18.1 | CR456460 |
| dJ366L4.C22.1 | dJ366L4.C22.1 | pGEM.dJ366L4.1.V2 | CR456461 |
| dJ366L4.C22.2 | dJ366L4.C22.2 | pGEM.dJ366L4.2.V3 | CR456462 |
| dJ37E16.C22.7 | dJ37E16.C22.7 | pGEM.dJ37E16.7 | CR456463 |
| dJ402G11.C22.5 | dJ402G11.C22.5 | pGEM.dJ402G11.5.V2 | CR456464 |
| dJ402G11.C22.7 | dJ402G11.C22.7 | pGEM.dJ402G11.7.V5 | CR456465 |
| dJ402G11.C22.8 | dJ402G11.C22.8 | pGEM.dJ402G11.8 | CR456466 |
| dJ402G11.C22.9 | dJ402G11.C22.9 | pGEM.dJ402G11.9.V2 | CR456467 |
| dJ408N23.C22.2 | dJ408N23.C22.2 | pGEM.dJ408N23.2.V2 | CR456468 |
| dJ430N8.C22.1 | Em:AC004542.C22.1 | pGEM.dJ430N8.1 | CR456469 |
| dJ439F8.C22.1 | dJ439F8.C22.1 | pGEM.dJ439F8.1 | CR456470 |
| dJ466N1.C22.4 | dJ466N1.C22.4 | pGEM.dJ466N1.4.V3 | CR456471 |
| dJ494G10.1 | dJ494G10.C22.1 | pGEM.dJ494G10.1 | CR456472 |
| dJ508I15.2 | dJ508I15.C22.2 | pGEM.dJ508I15.2 | CR456473 |
| dJ508I15.4 | dJ508I15.C22.4 | pGEM.dJ508I15.4 | CR456474 |
| dJ508I15.C22.5 | dJ508I15.C22.5 | pGEM.dJ508I15.5 | CR456475 |
| dJ549K18.C22.1 | dJ549K18.C22.1 | pGEM.dJ549K18.1 | CR456476 |
| dJ569D19.1 | dJ569D19.C22.1 | pGEM.dJ569D19.1.V2 | CR456477 |
| dJ579N16.3 | dJ579N16.C22.3 | pGEM.dJ579N16.3 | CR456478 |
| dJ579N16.C22.4 | dJ579N16.C22.4 | pGEM.dJ579N16.4 | CR456479 |
| dJ671O14.C22.2 | dJ671O14.C22.2 | pGEM.dJ671O14.2 | CR456480 |
| dJ742C19.2 | dJ742C19.C22.2 | pGEM.dJ742C19.2 | CR456481 |
| dJ756G23.3 | dJ756G23.C22.3 | pGEM.dJ756G23.3.V3 | CR456482 |
| dJ796I17.C22.2 | dJ796I17.C22.2 | pGEM.dJ796I17.2.V2 | CR456483 |
| dJ821D11.3 | dJ821D11.C22.3 | pGEM.dJ821D11.3 | CR456484 |
| dJ90G24.C22.6 | dJ90G24.C22.6 | pGEM.dJ90G24.6.V7 | CR456485 |
| DMC1 | dJ199H16.C22.1 | pGEM.DMC1 | CR456486 |
| DNAL4 | dJ327J16.C22.1 | pGEM.DNAL4 | CR456487 |
| DRG1 | bA247I13.C22.1 | pGEM.DRG1.V2 | CR456488 |
| EIF3S7 | dJ1119A7.C22.2 | pGEM.EIF3S7 | CR456489 |
| EWSR1 | bK984G1.C22.4 | pGEM.EWSR1 | CR456490 |
| FBXO7 | dJ149A16.C22.8.1 | pGEM.FBXO7.V2 | CR456491 |
| G22P1 | bK216E10.C22.1 | pGEM.G22P1.V2 | CR456492 |
| GGA1 | dJ437O22.C22.1 | pGEM.GGA1 | CR456493 |
| GGT1 | Em:AP000356.C22.4 | pGEM.GGT1 | CR456494 |
| GNAZ | Em:AC000029.C22.1 | pGEM.GNAZ.V4 | CR456495 |
| GNB1L | Em:AC000089.C22.2 | pGEM.GNB1L.V3 | CR456496 |
| GPR24 | bK229A8.C22.3 | pGEM.GPR24 | CR456497 |
| GRAP2 | dJ370M22.C22.1 | pGEM.GRAP2.V3 | CR456498 |
| GSTT1 | Em:AP000351.C22.10 | pGEM.GSTT1 | CR456499 |
| GSTT2 | Em:AP000350.C22.7 | pGEM.GSTT2 | CR456500 |
| GTPBP1 | dJ508I15.C22.3 | pGEM.GTPBP1 | CR456501 |
| H1F0 | dJ466N1.C22.1 | pGEM.H1F0 | CR456502 |
| HIRA | Em:AC000085.C22.1 | pGEM.HIRA | CR456503 |
| HMG2L1 | dJ510H16.C22.2 | pGEM.HMG2L1.V4 | CR456504 |
| HMOX1 | bK286B10.C22.2 | pGEM.HMOX1.V2 | CR456505 |
| IL2RB | dJ1170K4.C22.3 | pGEM.IL2RB.V7 | CR456506 |
| KCNJ4 | dJ434P1.C22.2 | pGEM.KCNJ4 | CR456507 |
| KCNMB3L | Em:AP000547.C22.5 | pGEM.KCNMB3L | CR456508 |
| KDELR3 | dJ434P1.C22.3 | pGEM.KDELR3 | CR456509 |
| LARGE | bK282F2.C22.1 | pGEM.LARGE | CR456510 |
| LGALS1 | dJ37E16.C22.3 | pGEM.LGALS1 | CR456511 |
| LGALS2 | dJ1177I5.C22.3 | pGEM.LGALS2.V3 | CR456512 |
| LIMK2 | Em:AC002073.C22.1c | pGEM.LIMK2 | CR456513 |
| MAPK11 | dJ402G11.C22.2 | pGEM.MAPK11 | CR456514 |
| MAPK12 | dJ402G11.C22.1 | pGEM.MAPK12 | CR456515 |
| MB | fF62D4.C22.1 | pGEM.MB | CR456516 |
| MCM5 | bK286B10.C22.3 | pGEM.MCM5 | CR456517 |
| MFNG | bK390B3.C22.1.1 | pGEM.MFNG | CR456518 |
| MGAT3 | dJ1104E15.C22.1 | pGEM.MGAT3 | CR456519 |
| MIF | Em:AP000350.C22.3 | pGEM.MIF | CR456520 |
| MIL1 | Em:AC006285.C22.5 | pGEM.MIL1 | CR456521 |
| MKL1 | dJ1042K10.C22.3 | pGEM.MKL1 | CR456522 |
| MPST | cE146D10.C22.2 | pGEM.MPST | CR456523 |
| MSE55 | dJ1177I5.C22.2 | pGEM.MSE55.V2 | CR456524 |
| MTMR3 | dJ394A18.C22.1 | pGEM.MTMR3.V2 | CR456525 |
| MYH9 | dJ68O2.C22.2 | pGEM.MYH9 | CR456526 |
| NAGA | bK250D10.C22.5 | pGEM.NAGA | CR456527 |
| NCF4 | bK833B7.C22.1 | pGEM.NCF4.V2 | CR456528 |
| NDUFA6 | dJ257I20.C22.3 | pGEM.NDUFA6 | CR456529 |
| NF2 | Em:AC005529.C22.1 | pGEM.NF2 | CR456530 |
| NHP2L1 | bK216E10.C22.2 | pGEM.NHP2L1 | CR456531 |
| NLVCF | Em:AC000068.C22.1 | pGEM.NLVCF | CR456532 |
| NUP50 | bK217C2.C22.1 | pGEM.NUP50 | CR456533 |
| OSM | Em:AC004264.C22.1 | pGEM.OSM.V6 | CR456534 |
| P2RXL1 | Em:AC002472.C22.1 | pGEM.P2RXL1 | CR456535 |
| PACSIN2 | dJ323M22.C22.1 | pGEM.PACSIN2.V3 | CR456536 |
| PCQAP | Em:AC004033.C22.1 | pGEM.PCQAP.V2 | CR456537 |
| PDGFB | cN10C3.C22.1 | pGEM.PDGFB.V2 | CR456538 |
| PES1 | Em:AC005006.C22.1 | pGEM.PES1 | CR456539 |
| PISD | dJ858B16.C22.2 | pGEM.PISD.V4 | CR456540 |
| PITPNB | dJ353E16.C22.1 | pGEM.PITPNB | CR456541 |
| PK1.3 | Em:AC005529.C22.7 | pGEM.PK1.3 | CR456542 |
| PLA2G6 | bK228A9.C22.1 | pGEM.PLA2G6.V4 | CR456543 |
| PMM1 | dJ347H13.C22.3 | pGEM.PMM1 | CR456544 |
| PNUTL1 | Em:AC000093.C22.1 | pGEM.PNUTL1 | CR456545 |
| POLR2F | dJ1039K5.C22.5 | pGEM.POLR2F | CR456546 |
| PPARA | dJ695O20A.C22.1 | pGEM.PPARA | CR456547 |
| PPIL2 | Em:AP000553.C22.2 | pGEM.PPIL2 | CR456548 |
| PRAME | Em:D87011.C22.1 | pGEM.PRAME | CR456549 |
| PRKCABP | dJ1039K5.C22.1 | pGEM.PRKCABP | CR456550 |
| PSCD4 | dJ63G5.C22.1 | pGEM.PSCD4 | CR456551 |
| PVALB | fF24E5.C22.1 | pGEM.PVALB | CR456552 |
| RAB36 | Em:AC000102.C22.1 | pGEM.RAB36.V8 | CR456553 |
| RABL2B | Em:AC002055.C22.1 | pGEM.RABL2B.V2 | CR456554 |
| RAC2 | dJ151B14.C22.2 | pGEM.RAC2 | CR456555 |
| RANBP1 | Em:AC006547.C22.1 | pGEM.RANBP1.V3 | CR456556 |
| RANGAP1 | dJ756G23.C22.2 | pGEM.RANGAP1 | CR456557 |
| RAYL | cE132D12.C22.1 | pGEM.RAYL | CR456558 |
| RBM9 | dJ41P2.C22.1 | pGEM.RBM9.V2 | CR456559 |
| RBX1 | bA554C12.C22.1 | pGEM.RBX1 | CR456560 |
| RFPL1S | Em:AC002059.C22.2 | pGEM.RFPL1S | CR456561 |
| RFPL1 | Em:AC002059.C22.1 | pGEM.RFPL1.V2 | CR456562 |
| RFPL2 | dJ90G24.C22.1 | pGEM.RFPL2.V2 | CR456563 |
| RFPL3 | dJ149A16.C22.2 | pGEM.RFPL3 | CR456564 |
| RFPL3S | dJ149A16.C22.3 | pGEM.RFPL3S.V2 | CR456565 |
| RPL3 | dJ333H23.C22.1.1 | pGEM.RPL3 | CR456566 |
| RTDR1 | Em:AC000029.C22.2 | pGEM.RTDR1 | CR456567 |
| SCA10 | bK941F9.C22.2 | pGEM.SCA10 | CR456568 |
| SCO2 | Em:U62317.C22.3 | pGEM.SCO2 | CR456569 |
| SDF2L1 | Em:AP000553.C22.4 | pGEM.SDF2L1 | CR456570 |
| SEC14L2 | Em:AC004832.C22.6 | pGEM.SEC14L2 | CR456571 |
| SEPT3 | bK250D10.C22.3 | pGEM.SEPT3.V2 | CR456572 |
| SERPIND1 | Em:AC007308.C22.3 | pGEM.SERPIND1.V2 | CR456573 |
| SEZ6L | dJ268D13.C22.1b | pGEM.SEZ6L.V2 | CR456574 |
| SF3A1 | Em:AC004997.C22.3 | pGEM.SF3A1 | CR456575 |
| SH3BP1 | dJ37E16.C22.2 | pGEM.SH3BP1 | CR456576 |
| SLC25A17 | dJ362J20.C22.1 | pGEM.SLC25A17 | CR456577 |
| SLC25A18 | Em:AC004019.C22.5 | pGEM.SLC25A18 | CR456578 |
| SLC5A1 | cB1E7.C22.1 | pGEM.SLC5A1.V4 | CR456579 |
| SLC7A4 | Em:AC002472.C22.5 | pGEM.SLC7A4 | CR456580 |
| SMARCB1 | Em:AP000349.C22.2 | pGEM.SMARCB1 | CR456581 |
| SNAP29 | Em:AC007308.C22.2 | pGEM.SNAP29 | CR456582 |
| SNRPD3 | Em:AP000356.C22.7 | pGEM.SNRPD3 | CR456583 |
| SOX10 | dJ1039K5.C22.4 | pGEM.SOX10 | CR456584 |
| SSTR3 | dJ151B14.C22.3 | pGEM.SSTR3.V2 | CR456585 |
| ST13 | dJ408N23.C22.1 | pGEM.ST13.V4 | CR456586 |
| STK22A | Em:AC004471.C22.3 | pGEM.STK22A | CR456587 |
| SULT4A1 | dJ388M5.C22.3 | pGEM.SULT4A1.V3 | CR456588 |
| SYN3 | cN28H9.C22.1 | pGEM.SYN3.V3 | CR456589 |
| SYNGR1 | dJ333H23.C22.2.2 | pGEM.SYNGR1 | CR456590 |
| TCN2 | Em:AC005006.C22.3 | pGEM.TCN2.V2 | CR456591 |
| TEF | dJ979N1.C22.1 | pGEM.TEF | CR456592 |
| TIMP3 | dJ309I22.C22.1 | pGEM.TIMP3.V2 | CR456593 |
| TOB2 | bK223H9.C22.1 | pGEM.TOB2 | CR456594 |
| TOM1L1 | dJ510H16.C22.1 | pGEM.TOM1L1 | CR456595 |
| TOP3B | Em:D87012.C22.1 | pGEM.TOP3B.V4 | CR456596 |
| TPST2 | bK445C9.C22.4 | pGEM.TPST2.V3 | CR456597 |
| TST | cE146D10.C22.1 | pGEM.TST | CR456598 |
| TTLL1 | dJ323M22.C22.2.a | pGEM.TTLL1.V4 | CR456599 |
| TUBA8 | Em:AC008101.C22.1 | pGEM.TUBA8.V2 | CR456600 |
| TXN2 | dJ1119A7.C22.1 | pGEM.TXN2 | CR456601 |
| Em:U51561.C22.2 | Em:U51561.C22.2 | pGEM.Em:U51561.2 | CR456602 |
| Em:U62317.C22.15 | Em:U62317.C22.15 | pGEM.Em:U62317.15.V5 | CR456603 |
| Em:U62317.C22.2 | Em:U62317.C22.2 | pGEM.Em:U62317.2 | CR456604 |
| Em:U62317.C22.9 | Em:U62317.C22.9 | pGEM.Em:U62317.9 | CR456605 |
| UBE2L3 | Em:AP000553.C22.1 | pGEM.UBE2L3.V2 | CR456606 |
| UFD1L | Em:AC000068.C22.2 | pGEM.UFD1L | CR456607 |
| UPK3 | bK268H5.C22.2 | pGEM.UPK3.V4 | CR456608 |
| VPREB1 | Em:D88270.C22.1 | pGEM.VPREB1 | CR456609 |
| VPREB3 | Em:AP000348.C22.2 | pGEM.VPREB3.V2 | CR456610 |
| XBP1 | bK292E10.C22.1 | pGEM.XBP1.V2 | CR456611 |
| YWHAH | cN44A4.C22.1 | pGEM.YWHAH.V2 | CR456612 |
| ZNF278 | Em:AC005003.C22.3 | pGEM.ZNF278 | CR456613 |
| ZNF279 | Em:D87009.C22.3 | pGEM.ZNF279 | CR456614 |
| ZNF70 | Em:AP000348.C22.1 | pGEM.ZNF70.V4 | CR456615 |
| ZNF74 | Em:AC005500.C22.2 | pGEM.ZNF74.V3 | CR456616 |
