# Supplementary material for: A genome annotation-driven approach to cloning the human ORFeome
Source: Genome Biol. 2004 Sep 30;5(10):R84. doi: 10.1186/gb-2004-5-10-r84 (PMC545604; doi:10.1186/gb-2004-5-10-r84)
Supplement: Additional data file 2 — Enzymes and templates used to amplify ORFs [file gb-2004-5-10-r84-s2.doc]

Supplementary table 2 Enzymes and templates used to amplify ORFs

| Round 1  Enzyme and annealing temperature | Round 2  Enzyme and annealing temperature | Universal cDNA | Other cDNA | Total |
| --- | --- | --- | --- | --- |
| KOD 60^0^ | KOD 60^0^ | 211 | 19 | 230 |
| KOD 60^0^ | KOD 65^0^ | 11 | 0 | 11 |
| KOD 65^0^ | KOD 60^0^ | 1 | 0 | 1 |
| *Pfu*-turbo 60^0^ | KOD 65^0^ | 4 | 0 | 4 |
| *Pfu*-turbo 60^0^ | *Pfu*-turbo 60^0^ | 6 | 1 | 7 |
| KOD65^0^ | KOD 65^0^ | 8 | 0 | 8 |
| *Pfu*-turbo 60^0^ | KOD 65^0^ | 5 | 0 | 5 |
| *Pfu* 60^0^ | *Pfu* 60^0^ | 0 | 9 | 9 |
| KOD 55^0^ | KOD 55^0^ | 2 | 0 | 2 |
| KOD 60^0^ | KOD 55^0^ | 1 | 0 | 1 |
|  |  | 249 | 29 | 278 |
