# Supplementary material for: A genome annotation-driven approach to cloning the human ORFeome
Source: Genome Biol. 2004 Sep 30;5(10):R84. doi: 10.1186/gb-2004-5-10-r84 (PMC545604; doi:10.1186/gb-2004-5-10-r84)
Supplement: Additional data file 3 — The sequence variation between ORF clone and genomic sequence [file gb-2004-5-10-r84-s3.doc]

Supplementary table 3 Sequence variation between ORF clone and genomic sequence

| cDNA clone | Base in ORF | dbSNP or dbEST identifier | Codon position of SNP | Codon in genomic | Codon in cDNA | Amino acid in genomic | Amino acid in cDNA | Type on SNP |
| --- | --- | --- | --- | --- | --- | --- | --- | --- |
| pGEM.ACO2 | 670 | rs11554472 | codon_pos=1 | CTG | TTG | Leu | Leu | SYN |
| pGEM.ACR.V3 | 886 | rs5771002 | codon_pos=1 | ATG | GTG | Met | Val | NON-SYN |
| pGEM.ADORA2A.V2 | 1083 | rs5751876 | codon_pos=3 | TAT | TAC | Tyr | Tyr | SYN |
| pGEM.APOL3 | 351 | rs3827346 | codon_pos=3 | AAT | AAC | Asn | Asn | SYN |
| cDNA pGEM.APOL4.V2 | 194 | rs2227168 | codon_pos=2 | CGT | CAT | Arg | His | NON-SYN |
| cDNA pGEM.APOL4.V2 | 219 | rs2227167 | codon_pos=3 | TCT | TCC | Ser | Ser | SYN |
| cDNA pGEM.APOL4.V2 | 426 | rs6000174 | codon_pos=3 | CTT | CTC | Leu | Leu | SYN |
| cDNA pGEM.APOL4.V2 | 482 | rs6000173 | codon_pos=2 | GCA | GAA | Ala | Glu | NON-SYN |
| cDNA pGEM.APOL4.V2 | 503 | rs6000172 | codon_pos=2 | TCG | TTG | Ser | Leu | NON-SYN |
| pGEM.ARFGAP1 | 1065 | rs3177212 | codon_pos=3 | AGT | AGG | Ser | Arg | NON-SYN |
| pGEM.ARFGAP1 | 1353 | rs738535 | codon_pos=3 | TCG | TCA | Ser | Ser | SYN |
| pGEM.ARFGAP1 | 1431 | rs3177213 | codon_pos=3 | AGT | AGC | Ser | Ser | SYN |
| pGEM.bA494O16.1.V3 | 525 | rs910796 | codon_pos=3 | AGT | AGC | Ser | Ser | SYN |
| pGEM.bA494O16.1.V3 | 573 | rs910797 | codon_pos=3 | CCA | CCG | Pro | Pro | SYN |
| pGEM.bA494O16.1.V3 | 1128 | rs910798 | codon_pos=3 | CCA | CCG | Pro | Pro | SYN |
| pGEM.bA494O16.1.V3 | 1258 | rs910799 | codon_pos=1 | ATA | GTA | Ile | Val | NON-SYN |
| pGEM.bA494O16.1.V3 | 1332 | rs910800 | codon_pos=3 | GGC | GGT | Gly | Gly | SYN |
| pGEM.bA494O16.1.V3 | 2826 | rs5770755 | codon_pos=3 | GCT | GCC | Ala | Ala | SYN |
| pGEM.bK268H5.4 | 385 | rs11556482 | codon_pos=1 | GTG | CTG | Val | Leu | NON-SYN |
| pGEM.bK268H5.4 | 420 | * | codon_pos=3 | AGG | AGA | Arg | Arg | SYN |
| pGEM.bK268H5.4 | 716 | rs6007594 | codon_pos=2 | CGT | CAT | Arg | His | NON-SYN |
| pGEM.bK747E2.1 | 102 | rs6005977 | codon_pos=3 | TCA | TCG | Ser | Ser | SYN |
| pGEM.bK747E2.1 | 129 | * | codon_pos=3 | GCC | GCT | Ala | Ala | SYN |
| pGEM.bK1048E9.4 | 686 | rs713998 | codon_pos=2 | GAG | GGG | Glu | Gly | NON-SYN |
| pGEM.bK1048E9.4 | 1327 | rs2014410 | codon_pos=1 | CTC | GTC | Leu | Val | NON-SYN |
| pGEM.bK1048E9.4 | 1654 | rs5752330 | codon_pos=1 | GTG | ATG | Val | Met | NON-SYN |
| pGEM.bK1048E9.4 | 1816 | rs1894706 | codon_pos=1 | CAT | TAT | His | Tyr | NON-SYN |
| pGEM.bK1048E9.4 | 1875 | rs1894704 | codon_pos=3 | CAG | CAT | Gln | His | NON-SYN |
| pGEM.CECR1 | 159 | rs362129 | codon_pos=3 | AAT | AAC | Asn | Asn | SYN |
| pGEM.COMT | 609 | rs165631 | codon_pos=3 | CTT | CTC | Leu | Leu | SYN |
| pGEM.CRYBA4.V3 | 204 | rs5761637 | codon_pos=3 | TTT | TTC | Phe | Phe | SYN |
| pGEM.CRYBB2 | 483 | rs8140949 | codon_pos=3 | GGG | GGA | Gly | Gly | SYN |
| pGEM.CRYBB3 | 337 | rs9608378 | codon_pos=1 | CAT | GAT | His | Asp | NON-SYN |
| pGEM.CSF2RB.V2 | 1962 | rs131840 | codon_pos=3 | CCA | CCG | Pro | Pro | SYN |
| pGEM.CYP2D6.V2 | 31 | rs769258 | codon_pos=1 | GTG | ATG | Val | Met | NON-SYN |
| pGEM.CYP2D6.V2 | 733 | EST_CK032848 | codon_pos=1 | CGC | TGC | Arg | Cys | NON-SYN |
| pGEM.CYP2D6.V2 | 1304 | EST_CK032849 | codon_pos=2 | AGC | ACC | Ser | Thr | NON-SYN |
| pGEM.DDX17 | 1935 | * | codon_pos=3 | CCC | CCA | Pro | Pro | SYN |
| pGEM.DGCR6 | 15 | rs408469 | codon_pos=3 | GCC | GCG | Ala | Ala | SYN |
| pGEM.DGCR6 | 108 | rs409155 | codon_pos=3 | CCG | CCC | Pro | Pro | SYN |
| pGEM.dJ102D24.2 | 516 | rs1022477 | codon_pos=3 | TTG | TTA | Leu | Leu | SYN |
| pGEM.dJ102D24.2 | 585 | rs1022478 | codon_pos=3 | TTC | TTG | Phe | Leu | NON-SYN |
| pGEM.dJ102D24.2 | 785 | rs2072770 | codon_pos=2 | CGG | CAG | Arg | Gln | NON-SYN |
| pGEM.dJ127B20.3.V2 | 480 | rs6519902 | codon_pos=3 | GCC | GCT | Ala | Ala | SYN |
| pGEM.dJ151B14.4.V4 | 62 | rs229527 | codon_pos=2 | GGG | GTG | Gly | Val | NON-SYN |
| pGEM.dJ151B14.4.V4 | 125 | rs229526 | codon_pos=2 | CCT | CGT | Pro | Arg | NON-SYN |
| pGEM.dJ186O1.2.V2 | 306 | rs133383 | codon_pos=3 | GCC | GCT | Ala | Ala | SYN |
| pGEM.dJ222E13.1.V4 | 376 | rs137055 | codon_pos=1 | TGC | CGC | Cys | Arg | NON-SYN |
| pGEM.dJ402G11.5.V2 | 29 | rs2272846 | codon_pos=2 | ACC | AAC | Thr | Asn | NON-SYN |
| pGEM.dJ402G11.8 | 717 | rs138217 | codon_pos=3 | GCA | GCG | Ala | Ala | SYN |
| pGEM.dJ402G11.8 | 1293 | rs138222 | codon_pos=3 | CTC | CTT | Leu | Leu | SYN |
| pGEM.dJ402G11.9.V2 | 1113 | * | codon_pos=3 | CCT | CCC | Pro | Pro | SYN |
| pGEM.dJ402G11.9.V2 | 1263 | rs5771206 | codon_pos=3 | CTA | CTG | Leu | Leu | SYN |
| pGEM.dJ439F8.1 | 522 | rs2542040 | codon_pos=3 | TTT | TTC | Phe | Phe | SYN |
| pGEM.dJ549K18.1 | 1300 | rs2294918 | codon_pos=1 | AAG | GAG | Lys | Glu | NON-SYN |
| pGEM.dJ671O14.2 | 480 | rs2272942 | codon_pos=3 | GTC | GTT | Val | Val | SYN |
| pGEM.dJ742C19.2 | 418 | rs139300 | codon_pos=1 | AAG | GAG | Lys | Glu | NON-SYN |
| pGEM.dJ742C19.2 | 534 | rs139302 | codon_pos=3 | GAG | GAC | Glu | Asp | NON-SYN |
| pGEM.dJ756G23.3.V3 | 582 | rs9611519 | codon_pos=3 | CCC | CCT | Pro | Pro | SYN |
| pGEM.dJ1014D13.2 | 2424 | * | codon_pos=3 | AGG | AGA | Arg | Arg | SYN |
| pGEM.dJ1119A7.3 | 535 | rs760718 | codon_pos=1 | TTT | CTT | Phe | Leu | NON-SYN |
| pGEM.dJ1119A7.3 | 923 | rs2277841 | codon_pos=2 | AAC | AGC | Asn | Ser | NON-SYN |
| pGEM.dJ1170K4.2 | 72 | rs11704654 | codon_pos=3 | CCG | CCA | Pro | Pro | SYN |
| pGEM.Em:AC002073.2.V3 | 752 | rs2040533 | codon_pos=2 | ACC | AGC | Thr | Ser | NON-SYN |
| pGEM.Em:AC002472.7 | 618 | rs178266 | codon_pos=3 | GGT | GGC | Gly | Gly | SYN |
| pGEM.Em:AC002472.8 | 343 | rs426938 | codon_pos=1 | GCT | CCT | Ala | Pro | NON-SYN |
| pGEM.Em:AC004832.1.V2 | 234 | rs5753130 | codon_pos=3 | ACT | ACC | Thr | Thr | SYN |
| pGEM.Em:AC004997.11 | 181 | rs5749082 | codon_pos=1 | AGG | TGG | Arg | Trp | NON-SYN |
| pGEM.Em:AC005006.2 | 85 | rs2267161 | codon_pos=1 | GTG | ATG | Val | Met | NON-SYN |
| pGEM.Em:AC005006.4 | 483 | rs5997714 | codon_pos=3 | CCA | CCG | Pro | Pro | SYN |
| pGEM.Em:AC016026.2 | 2265 | rs9617630 | codon_pos=3 | CCG | CCA | Pro | Pro | SYN |
| pGEM.Em:AP000351.3 | 385 | rs140195 | codon_pos=1 | AAG | GAG | Lys | Glu | NON-SYN |
| pGEM.Em:AP000351.3 | 501 | rs6003994 | codon_pos=3 | TAT | TAC | Tyr | Tyr | SYN |
| pGEM.Em:AP000557.1.V3 | 858 | rs467181 | codon_pos=3 | GCT | GCC | Ala | Ala | SYN |
| pGEM.Em:AP000557.1.V3 | 1260 | rs128477 | codon_pos=3 | CAT | CAC | His | His | SYN |
| pGEM.Em:AP000557.3.V6 | 602 | rs377287 | codon_pos=2 | GGG | GAG | Gly | Glu | NON-SYN |
| pGEM.Em:AP000557.3.V6 | 640 | rs457751 | codon_pos=1 | GTC | ATC | Val | Ile | NON-SYN |
| pGEM.Em:AP000557.3.V6 | 995 | rs128478 | codon_pos=2 | ATG | ACG | Met | Thr | NON-SYN |
| pGEM.Em:AP000557.3.V6 | 1203 | rs2930768 | codon_pos=3 | TTC | TTT | Phe | Phe | SYN |
| pGEM.Em:AP000557.3.V6 | 1267 | rs2629350 | codon_pos=1 | CGC | TGC | Arg | Cys | NON-SYN |
| pGEM.Em:AP000557.3.V6 | 1636 | rs402724 | codon_pos=1 | CTC | TTC | Leu | Phe | NON-SYN |
| pGEM.Em:U51561.2 | 371 | rs738935 | codon_pos=2 | CTG | CAG | Leu | Gln | NON-SYN |
| pGEM.Em:U62317.9 | 123 | * | codon_pos=3 | CCT | CCC | Pro | Pro | SYN |
| pGEM.Em:U62317.9 | 856 | rs128941 | codon_pos=1 | CTG | TTG | Leu | Leu | SYN |
| pGEM.Em:U62317.9 | 1959 | rs6009975 | codon_pos=3 | CAA | CAG | Gln | Gln | SYN |
| pGEM.GGT1 | 504 | * | codon_pos=3 | TTC | TTT | Phe | Phe | SYN |
| pGEM.GNAZ.V4 | 309 | rs1805058 | codon_pos=3 | GAC | GAT | Asp | Asp | SYN |
| pGEM.GPR24 | 94 | rs133072 | codon_pos=1 | AAC | GAC | Asn | Asp | NON-SYN |
| pGEM.GPR24 | 246 | rs133073 | codon_pos=3 | AAC | AAT | Asn | Asn | SYN |
| pGEM.GSTT2 | 195 | rs364069 | codon_pos=3 | ACT | ACC | Thr | Thr | SYN |
| pGEM.GSTT2 | 363 | rs4822465 | codon_pos=3 | CCC | CCA | Pro | Pro | SYN |
| pGEM.GTSE1 | 1516 | rs140054 | codon_pos=1 | TGG | CGG | Trp | Arg | NON-SYN |
| pGEM.HIRA | 906 | * | codon_pos=3 | GTT | GTC | Val | Val | SYN |
| pGEM.KCNJ4 | 1002 | rs196059 | codon_pos=3 | TCA | TCG | Ser | Ser | SYN |
| pGEM.KCNMB3L | 375 | rs5747988 | codon_pos=3 | GCT | GCC | Ala | Ala | SYN |
| pGEM.KCNMB3L | 958 | rs11089254 | codon_pos=1 | TGG | CGG | Trp | Arg | NON-SYN |
| pGEM.LARGE | 435 | rs86487 | codon_pos=3 | GCC | GCT | Ala | Ala | SYN |
| pGEM.LIMK2 | 1209 | rs4141405 | codon_pos=3 | CTC | CTG | Leu | Leu | SYN |
| pGEM.MAPK11 | 507 | rs760748 | codon_pos=3 | TTT | TTC | Phe | Phe | SYN |
| pGEM.MAPK11 | 756 | rs2076139 | codon_pos=3 | TCA | TCG | Ser | Ser | SYN |
| pGEM.MAPK12 | 183 | rs2272857 | codon_pos=3 | CCT | CCC | Pro | Pro | SYN |
| pGEM.MAPK12 | 308 | EST_BI755093 | codon_pos=2 | ACG | ATG | Thr | Met | NON-SYN |
| pGEM.MAPK12 | 603 | rs2066781 | codon_pos=3 | TCT | TCC | Ser | Ser | SYN |
| pGEM.MB | 174 | rs7293 | codon_pos=3 | GCG | GCA | Ala | Ala | SYN |
| pGEM.MB | 204 | rs7292 | codon_pos=3 | ACC | ACT | Thr | Thr | SYN |
| pGEM.MIL1 | 771 | rs4488761 | codon_pos=3 | TCA | TCG | Ser | Ser | SYN |
| pGEM.MKL1 | 1806 | rs4821944 | codon_pos=3 | GCG | GCA | Ala | Ala | SYN |
| pGEM.MKL1 | 1942 | rs878756 | codon_pos=1 | AGC | GGC | Ser | Gly | NON-SYN |
| pGEM.MTMR3.V2 | 984 | rs2074204 | codon_pos=3 | GCC | GCT | Ala | Ala | SYN |
| pGEM.MYH9 | 3429 | rs710181 | codon_pos=3 | GCT | GCG | Ala | Ala | SYN |
| pGEM.P2RXL1 | 171 | rs2007013 | codon_pos=3 | GCT | GCG | Ala | Ala | SYN |
| pGEM.PK1.3 | 1573 | rs737976 | codon_pos=1 | GTT | ATT | Val | Ile | NON-SYN |
| pGEM.PK1.3 | 1735 | rs1049534 | codon_pos=1 | GTT | ATT | Val | Ile | NON-SYN |
| pGEM.PRAME | 19 | rs2266988 | codon_pos=1 | TGG | CGG | Trp | Arg | NON-SYN |
| pGEM.PSCD4 | 630 | rs2239822 | codon_pos=3 | TTT | TTC | Phe | Phe | SYN |
| pGEM.RABL2B.V2 | 201 | EST_BX103967 | codon_pos=3 | GGA | GGC | Gly | Gly | SYN |
| pGEM.RABL2B.V2 | 203 | EST_BX103967 | codon_pos=2 | AGG | AAG | Arg | Lys | NON-SYN |
| pGEM.RABL2B.V2 | 285 | rs2592656 | codon_pos=3 | CAC | CAT | His | His | SYN |
| pGEM.RABL2B.V2 | 307 | rs2595124 | codon_pos=1 | GTA | ATA | Val | Ile | NON-SYN |
| pGEM.RABL2B.V2 | 671 | EST_BX460068 | codon_pos=2 | GCG | GTG | Ala | Val | NON-SYN |
| pGEM.RANGAP1 | 1548 | rs71948 | codon_pos=3 | CTG | CTC | Leu | Leu | SYN |
| pGEM.RFPL1.V2 | 380 | rs3804076 | codon_pos=2 | ATG | ACG | Met | Thr | NON-SYN |
| pGEM.RFPL1S | 245 | rs3804076 | codon_pos=2 | CAT | CGT | His | Arg | NON-SYN |
| pGEM.RFPL2.V2 | 141 | rs136479 | codon_pos=3 | CTA | CTG | Leu | Leu | SYN |
| pGEM.RFPL2.V2 | 152 | rs136478 | codon_pos=2 | TGC | TAC | Cys | Tyr | NON-SYN |
| pGEM.RFPL2.V2 | 599 | rs136472 | codon_pos=2 | ACC | AGC | Thr | Ser | NON-SYN |
| pGEM.RFPL2.V2 | 603 | rs136471 | codon_pos=3 | ACG | ACC | Thr | Thr | SYN |
| pGEM.RFPL2.V2 | 700 | rs136470 | codon_pos=1 | AGT | GGT | Ser | Gly | NON-SYN |
| pGEM.RFPL2.V2 | 748 | rs136469 | codon_pos=1 | TTG | CTG | Leu | Leu | SYN |
| pGEM.RFPL2.V2 | 751 | rs136468 | codon_pos=1 | CGC | TGC | Arg | Cys | NON-SYN |
| pGEM.RFPL2.V2 | 772 | rs136467 | codon_pos=1 | GTT | ATT | Val | Ile | NON-SYN |
| pGEM.RPL3 | 111 | rs6509 | codon_pos=3 | CCG | CCA | Pro | Pro | SYN |
| pGEM.RTDR1 | 148 | rs4822360 | codon_pos=1 | TTG | CTG | Leu | Leu | SYN |
| pGEM.SCO2 | 59 | rs140523 | codon_pos=2 | CGG | CCG | Arg | Pro | NON-SYN |
| pGEM.SCO2 | 633 | rs12148 | codon_pos=3 | GCA | GCC | Ala | Ala | SYN |
| pGEM.SERPIND1.V2 | 263 | rs165867 | codon_pos=2 | CCC | CAC | Pro | His | NON-SYN |
| pGEM.SERPIND1.V2 | 1530 | rs4675 | codon_pos=3 | CAC | CAT | His | His | SYN |
| pGEM.SH3BP1 | 2067 | * | codon_pos=3 | CCT | CCG | Pro | Pro | SYN |
| pGEM.SLC7A4 | 83 | rs2072550 | codon_pos=2 | ACC | ATC | Thr | Ile | NON-SYN |
| pGEM.SLC7A4 | 1045 | rs2270384 | codon_pos=1 | GCC | ACC | Ala | Thr | NON-SYN |
| pGEM.SLC25A17 | 675 | * | codon_pos=3 | ACG | ACA | Thr | Thr | SYN |
| pGEM.SOX10 | 18 | * | codon_pos=3 | GAC | GAT | Asp | Asp | SYN |
| pGEM.TCN2.V2 | 776 | rs1801198 | codon_pos=2 | CGT | CCT | Arg | Pro | NON-SYN |
| pGEM.TIMP3.V2 | 249 | rs9862 | codon_pos=3 | CAT | CAC | His | His | SYN |
| pGEM.TOB2 | 234 | rs202641 | codon_pos=3 | GCG | GCA | Ala | Ala | SYN |
| pGEM.TOM1L1 | 1287 | rs743810 | codon_pos=3 | GGT | GGG | Gly | Gly | SYN |
| pGEM.TOM1L1 | 1401 | rs2071745 | codon_pos=3 | GGG | GGC | Gly | Gly | SYN |
| pGEM.TPST2.V3 | 270 | rs4822735 | codon_pos=3 | GCG | GCC | Ala | Ala | SYN |
| pGEM.UPK3.V4 | 549 | rs2075951 | codon_pos=3 | TCA | TCG | Ser | Ser | SYN |
| pGEM.UPK3.V4 | 858 | rs1057356 | codon_pos=3 | CAA | CAG | Gln | Gln | SYN |
| pGEM.ZNF70.V4 | 1221 | rs5759985 | codon_pos=3 | ATT | ATC | Ile | Ile | SYN |
| pGEM.ZNF278 | 522 | rs714909 | codon_pos=3 | CTC | CTT | Leu | Leu | SYN |
| pGEM.ZNF279 | 606 | rs2236730 | codon_pos=3 | TCG | TCA | Ser | Ser | SYN |
| pGEM.ZNF279 | 767 | rs2236729 | codon_pos=2 | GAA | GCA | Glu | Ala | NON-SYN |
| pGEM.ZNF279 | 1518 | rs11109 | codon_pos=3 | TCA | TCG | Ser | Ser | SYN |

* unconfirmed bases changes in dbSNP or dbEST which do not alter the amino acid
