# Supplementary material for: A genome annotation-driven approach to cloning the human ORFeome
Source: Genome Biol. 2004 Sep 30;5(10):R84. doi: 10.1186/gb-2004-5-10-r84 (PMC545604; doi:10.1186/gb-2004-5-10-r84)
Supplement: Additional data file 4 — The nested oligonucleotide primers designed for the 398 targeted genes [file gb-2004-5-10-r84-s4.doc]

Supplementary Table 4

Nested oligonucleotide primers designed for the 398 targeted genes

| **Locus** | **Innrer primer (sense)** | **Inner Primer (anti-sense)** | **Outer Primer (sense)** | **Outer Primer (anti-sense)** |
| --- | --- | --- | --- | --- |
| A4GALT | GATACCATGTCCAAGCCC | CAGGTTGGGGAGGTG | CCAGCCGGTTCCTG | CAGCTCCTCAACAGCC |
| ACO2 | TGTCAGTGCACAAAATGGC | GCATGTCTGGTCACACCATC | CGTCACTTTAATGCGACCTC | TTCAGCCACCAATGAAATGA |
| ACR | CAGGAGTATGGTTGAGATGCT | GTCAGATCAGGAGGTCGAGG | CAGGCAGTGCCAGGAGTATG | CCGCCTTATTGTAGGGTGAA |
| ADORA2A | ACTTGGCTCCTGTGAGGAAG | CTTCTCCCAACGTGACTGGT | GACCACATCTGATCCTTGGC | AGGGTCTCCTGGCACTCTCT |
| ADRBK2 | GCCAAAGCTCGCCAAC | AAATGTGACAAGGCAGGGAG | CGTCCAGGTCCGGAGTAAC | GCAATGCTTAGGGCAATCTG |
| ADSL | TCCAGTCCACCCTGGC | GCACCATGGGAAGAAGAAAG | GTTTCCGCTTCCGCTCTT | GCAGAGTCAACTTCAACGCTC |
| ALG12 | AGTGCTAACGGCTGGTGTCT | CCCAGTCCTTTGACTTGCTT | AGCCACTTTAGATTTGGGCA | GTTCTTTGGTGCTGAGAGCC |
| AP1B1 | CGGAAGCCTGGCTACAGATA | CCTGCGAGGAGGAAGATGT | CGGGAGCTATTGGGACCT | TGCTCTGGCTCTGTTTCCTT |
| APOL1 | GGCTGTGCTGTGTCCCTAAT | GCATATCTCTCCTGGTGGCT | AGACGCATAACTGGAGGTGG | CAACTTGGCGACAGAGCA |
| APOL2 | CAGCTGACACAGCAGACCTT | CTCTGGGGTCATTGGTCTTG | TCTGACTGCCTGAGACATGG | TGCTCAGCTACACAAATGCC |
| APOL3 | GAACCCTTCCAGTCAGGTCA | CTTGCCATCTGCATTAACCC | TGCCTTGGTGTGAGAGTGAG | ATTCCTGCCTTCTCCTTGGT |
| APOL4 | ATGACCAAGGAGGATGGGAT | GTGGAGCCAGACACCACATT | GCCTCAACATTCAGCAGAGG | TAACGCCAATGACAGACAGG |
| APOL5 | AGCATGCCATGTGGCAAACAAGG | ACCCCCAGTATTCTTCAGGG | TCTAAAGCATGCCATGTGGCAAAC | AGAAGTTTCCCTACCCCCAG |
| APOL6 | ACACAGATTTGCTGCCACAG | CCATTGTATGCCATGTCCTG | GAGCTCCGTGGAGAGAAGAA | TGACACCCAGACCTGTCTCA |
| ARFGAP1 | CACAGCTGACGATGGG | CTCCAGGAAATACACATC | CCGCTTTTCGTCGACTCTTA | CCGCCTGAGATGTGGTTACT |
| ARHGAP8 | GTCACCATGAGGACTCTCCG | GCTCGAAATATACAGAGTGTTCG | GCTCGGCTTCCTGCTCTC | TCTGAATTCATCTAATGGCTGGT |
| ARSA | GTATCGGAAAGAGCCTGCTG | ACGTTATCAGGCACAAACCC | CTCGAGAATCTGAAGGTGCC | TGCAAGTCTCCACTGGTGTT |
| ARVCF | GGACATGCTGATCTTCCCTC | CACGATCCAAGCCCTAAGAA | CTCCAGGAGCCAGAGCCT | CCTTCTGTCACTCGCTCACA |
| ATF4 | TCTAGAGAAGTCCCGCCTCA | TACAAGCACATTGACGCTCC | GGCAAATACAACTGCCCTGT | TGGAACACACAGCTACAGCA |
| ATP6E | CTTTCAAACCTAAACTCGAGCC | TCATATTACATGAAGCTTTCCACC | CTTTGCCGATTTCTCTCACC | AAGAACTGGAGGTGGGTCCT |
| bA247I13.C22.2 | CCGAGTGCAGAGCATTGTAG | AAAAGCCCATAAACCAACCC | GAGGCACCGAGTGCAGAG | GCTTCACACAGAGTGCTCCA |
| bA247I13.C22.4 | TTATGCGCGAGTACTGGTTG | GCCAAGATTCACAAGAAGGG | GTTCGCATAGGCAGAGAACC | AACACACTTTATTCAGTAATACAAACGA |
| bA494O16.C22.1 | TGAACCTAAGATACAGCCGGA | TGAGAGTGCCTGGTCTGATG | GCACAAATGAGCACTTGGAT | TAAGGCACGTGGAGACACTG |
| bA9F11.C22.1 | CTTTGCCTGGAAGGTCTCAG | GCAAGAAGGAGCAGAGATGG | GACCTAAAGTGTGACCCCGA | ACACAGAGCATCTCCAGTGAA |
| BCR | TCACCTGCCACCAGGG | GATGGCCTGATTAGCCAGAG | GAAGCGAGAGGCGAGGAG | CAGGAAGGTTCCCGCTCTAC |
| BID | TGTGAACCAGGAGTGAGTCG | ACAGCTGTGACCACATCGAG | CATAAGGAGGAAGCGGGTAG | CTCCGTCTACACTGGAAGCA |
| BIK | GAGAAATGTCTGAAGTAAGAC | GATAACAGCAGCAGG | GTGGCTTACAGACGCTGCC | AACCTCAGCAGTGTTCCAGC |
| bK1048E9.C22.3 | AGTATCTGAGGATCGGTGGC | CGACTCCAGCATGGGAAATA | GACCGACGGCACTTGATAAT | AACAACACCGGTAGGGACTG |
| bK1048E9.C22.4 | GGGATAGAGGAACGGTCACA | ACATCCCAATCTGCAAAAGG | AACCTGCAAGGAAGGGAAGT | GGCTCAGTATGGCAGAGAGG |
| bK1048E9.C22.5 | AGACCAATGGCTGCGGCCG | AGTCTCTGGTGGCAGCTTCT | CGTCAGAGACCAATGGCTG | CCATAGCAGCCTTCCCTTTA |
| bK1191B2.C22.3 | CAGGTGTCCGACCATGAGC | TCCTCAGGAGGACAGAGGG | CTCGGTCGCCACGGTAAC | GGGGTCATCGCATTTGAG |
| bK126B4.2 | CATTCGCCAGACGAGAGC | ACTTCCACAAACTGGAACCG | TGGAACTGCAGGATACACTCC | CAGTGTTGAGTCTGGGAGCA |
| bK126B4.3 | CTGACATTCGCTCCCACTTT | CAGGACTGCTGAAGCACAAG | TGCATGAGTGGAAAGCTGAG | ACAGAACGGATTCATCCAGG |
| bK150C2.1 | GGAGGACACAGACCAGGAAC | GAGATGGTGGTGAACGGTCT | TGGAATCTTCCCTGGACAAG | ATACTGCTTTGCTGGCGTCT |
| bK150C2.2 | GACAAGCGTATCTAAGAGGCTGA | GAGATGGTGGTGAACGGTCT | AAAGAGCGGGACAGGGAC | GTGTCTGTGAGCAGCTGGAG |
| bK150C2.3 | AAGGACGCTGTAAGCAGGAA | CATGAGCAGGAGGCTAGAGG | GCCCTGGGAGGTCACTTTA | CCTCTGCAGGAAGAGTCTGG |
| bK150C2.4 | GGAGGTCACTTTAGGGAGGG | TGCACCAAGACATGAGCTTC | CAGAGCGGCCTGTCTTTATC | CCCATCTCAGTCCTGCTGTT |
| bK150C2.9 | CAGGAAGTGAAACCACAGCA | AGCATGAGCAGGAGGCTAGA | TAAGGAGGGCTGTCCAACTG | CACTATGGAATGGCCCTGAG |
| bK175E3.C22.6 | CTACACCACCGGCCTCAC | CCATACAGTTCCCAATTTCCA | GAGGTGGTGCTGTTCGAGT | AGGCAAAGGACTCGGAGAAT |
| bK216E10.C22.6 | TAGAGAGACCCAGGCTCCAA | CCAGGATATTTGTGCCATCC | TGTGCACTTGGAAGTCTTGG | TTGTTCCTGGATCTCAGCCT |
| bK221G9.C22.4 | CCATGGAAGTGTGTGGACAG | AGCGTTGTGCAGGAAGAAAC | TGCTGGGACGAGTCTTCTTT | CTCCTTTCCCAGCCTGAAGT |
| bK223H9.2 | GTGGCCGGCTTAGTTAGGAG | CGATATGGTGTGCCAGAGTG | GGTTAAAGGTCGGACGGAAG | CGTCGCTTCACAGTCAGTTC |
| bK250D10.2 | ACTCAGGGCGGTTTGAAAG | GTCAAGCCCTGACTGGACAT | AAGGAGATAAAGGTTCGGTTCC | TGCAAGAGTGAGTGTGGGAG |
| bK250D10.C22.6 | AAGCAAGATGGCGGTGAAT | GAACATGGGAGCATCTGGTT | GAGGCCCGAAGCAAGATG | GGGAAACCTTATCCCTGCTC |
| bK250D10.C22.7 | ACCATGAGGCGAGGGCCCCGGAG | ATGTGCATGCTGGAGTGAAG | GCACCATGAGGCGAGGG | CTGCAGAGTTAGCCTGGTCC |
| bK250D10.C22.8 | ACTCTGCCCTCCACTGGACT | GTTGGGACCCACTATGGACA | CTTGCTCTCCGTGTCAAGC | GGCTAGGAAACTCCAGCAGA |
| bK268H5.C22.1 | CACATGCTGCGTGCCATAG | GGTGGACAGGTAGGCACTTG | GAGGCCGAGGAGCGCTC | CGGCATCTCTAGTCCTCTTTG |
| bK268H5.C22.4 | CCGCAGAATTCACAGATGG | TTTTCAAGTTGCAGGTTTTACTG | CTCTCGCTCTCGCTTCTAGC | TTGGGCACTGTGGTTACAGA |
| bK29F11.C22.1 | GGCGGCGGAGATGGGG | TTCCCAGTTTGTGAGCAGG | CAGCCTGGCGGACGAG | TGACCTGCGTGGAAATCATA |
| bK445C9.C22.6 | ACCTAGGGACCACCAAGGAT | CCCTCTTAGGGGCAAGTCTC | CGGATGGTGAAGCTGAATTT | CAGAGAACTCCTTTGCCAGG |
| bK57G9.C22.2 | ACGGCCCATGGCGCCGCCAG | CTGTAGGAGGAGGGTTTCCC | ACTGACGGCCCATGGCGC | GATGAAGCAGGAGGAATCCA |
| bK747E2.C22.1 | AAGGCAACTGAAATATGCAC | TCCCATGAGTTCTTGTTCCT | TCCCCTTACTAAGCTAGCGAAA | TGCAAAGTTGTGTTTATTTTTCAGA |
| bK963H5.C22.1 | CATGGACACAGCCTAGCAGA | CTCTGACTCCAATGCAGGG | CCGCCTGAAGGAGAGAGTTT | TGTCACTCACAGGGCAGAAA |
| bK984G1.1 | CTGGTGCAGCATGGGC | CACCACCCTCAGCCTCTCT | GGGAGGACAGGCTGGG | AGAAGGGACCCTGAGGACAT |
| BRD1 | GACTCACAGAGGGAAGCCTG | ACACTATGGACAAGACCCGC | TACCCAGTGTCTGGAGGGTC | CAGACGGAGATGGGTTCCTA |
| BZRP | GCAGCAGCCATGGC | CCAGTGGTCATGAAAGC | CCTGGCTAACTCCTGCCA | AAGGCCCTGACAGACTAGCA |
| C22orf2 | CACTGGCTTTGCTTTCATCA | TCACCTTCTATTGCCAACCC | GGCTACAGAGTCCTTGCTGG | GTGTTTGCGAGCTTGTGTGT |
| C22orf3 | TGTCTTTCCATTCTGCCCTC | CACTGTGGCCCTCTTTAGACA | AAACTGAGGCCCAAGGAAAT | CAAGGAGGTCCAGGTTGAAA |
| C22orf4 | GTCTGAGGGATGAGGAGGG | AAAGGCTGTCCCTGTCTTTG | AGCTTCTCGGCTCTAGGCTC | CAAGGACATTGCAGGGAGAG |
| C22orf5 | TCTGCAAAGGTTTCCCTCAG | AATAAATAAAGGCGGCGTGA | GGCTCCTGAGGCTAGCTTGT | TAAGCCTTGCTCCCAGTGTC |
| CACNA1I | GTCTCTAACCTGGACGACCC | CCCAGGTGTGGACGAAGTAT | TCAGCTGATCCTGAATTGGC | CTAACCCAGACTCCGCTCAG |
| CACNG2 | CAGAAGTCGGTTGGGTGTTT | TTTGGAAGGTCTCCCAGC | ACCCTCACACACACTCTCCC | TCCCACATTTCCTGTTGTTT |
| cB42E1.1 | ACCGTTAGCTCGAGGCG | GAAGTCCTGGCTTTTGGTGA | TAGCTGGCGGGACCGTTA | TTCCATGAGCCTCCTGTACC |
| cB5E3.C22.2 | CACCTCCGACAAGGACAGAT | AGACCATTGCAGCCACCTAC | CCGTACAGCTATGTGGGACA | ACATGAGCTGGTTCTTTGGG |
| CBX6 | GCTGAGCAAGATGGAGCTGT | GGGTGGGAGCAAGAGTATGA | GTATTATGGGCTGTGGGTGC | AACACCGAGCCATTTGAGAC |
| CBX7 | CGCCAGCCCCAGCATC | GTGGTGGGAGAGTAGTGGGA | ACGTTGCGCAGGTTCAAA | ATGATAATGGTGGTGTCCCG |
| CDC45L | GGCTATGTTCGTGTCCGATT | GCCAGTTACATAAATAAGAAGGTCA | CGGGCTCTTGGTACCTCA | TGGCTTCTACATCTCAAATCATGT |
| cE81G9.C22.2 | GATCTGCACTTCTGGGCTTC | GAAGACACCTCTACTGTGTCAAAA | AGTTCTGCAGTGGGGATCTG | GAGCCTTTCCTTATTGTTAGCAC |
| CECR1 | TCCATCTGAGCCCTTTCCTA | GGTCAATGTCACTGTGGCTG | AACAGCCCTCAGTTTCTGGA | AATGGCCAGAGACAGGAGAA |
| CELSR1 | GCCATGGCGCCGCCGCCGCCGCCCGTG | CTCCTGGGAGAACCAAGACC | TCCGGGCCATGGCGCCGCCG | GCACTTGTCACCAGGTCTGA |
| CHEK2 | CAGGTTTAGCGCCACTCTG | CCACGGAGTTCACAACACAG | GGCTGAGGGTGGAGTTTGTA | TTCAAACCACGGAGTTCACA |
| CHKL | GAGAGACCCGAGTATGACCG | CTTCTGCTCGTTGTTCCTCC | GAGAGACCCGAGTATGACCG | CTTCTGCTCGTTGTTCCTCC |
| CLDN5 | AGAGGCTCTGTGATTGGCTG | GGCTAGTGGCAGGAGAAGGT | CAAGAGCCGTTGTTTCCCTA | TCATTCCGTCTGTTAAGGGC |
| CLTCL1 | CATGGCGCAGATCCTCC | AGTGCAATCAGCTGGGTCTC | GAGGTCCCGCACCAGC | TGTCGGCTAAAGCTGGTCTT |
| cN121E8.C22.1 | ATATCCATGTGGCTTCACCC | CACTGTGTGGTCCTGTCCTG | CACTTGTTGTGCACCTTGCT | GGTTCAATGGCCCAAAGATA |
| cN5H6.C22.1 | ACATGGCCGCAGCCTC | ACGCCACCACTGAGGTTCT | AGAGCTCGCGGTGGACTC | ATGGGACAGCAACACAATGA |
| cN5H6.C22.4 | ACTGGTAGACGGGTCAGTGG | CAGGCCAAACTCTTTCCAAG | ATGTGTTGGAGAGAGGTGGG | GGCAGAAATGAATCTGCCAT |
| COMT | AGAGGTGCTTTGAAGATGCC | CAGGCTGGGTGAGAGAGC | GTCGCGGGAGAGAAATAACA | CACGTCTGGCACCTTCAGTA |
| CPT1B | GGGTATGAAGACGACCCTGA | AATTCCCTTCCTGCTCCAAC | AGTGGTTAGCGTTCATGCTG | ACCTCTGTGGTCTGAGCTGG |
| CRKL | GAGCCGAGAGGAAAGTGCT | CAGAACAACAAAGCAGCAGG | GAGTCACTGGAGGCACCC | CAATGCAGTGTGACTTGCCT |
| CRYBA4 | GACATGTTCCCTGGGCCTATC | GGAGGCAGAAGGAGAGAACA | AGATCTGACATGTTCCCTG | TGCTGGGTTCACACAGGTTA |
| CRYBB1 | CAGGAAGTAGCAAGTCCCCA | GGGCAAGGTAGCAGAGTGAG | GGTCGGACTCTAGTCACCCA | CGAGGAAGTCACATCCCAGT |
| CRYBB2 | ACAGTCCACCATGGCCTCA | GAGGTCTGGAGGGTTCCTG | GTCACTGGTCATTCCTGCAC | TGCAAGTCACACTTTATTCACTCTC |
| CRYBB3 | TACAGCAACAGCCAGAGGTG | TCTGGGTCCTTGAAGTCTGG | GTGGCTCCTCTGTTCTTCCC | CCCAGGCTTTATTGAGCAGA |
| CSF2RB | CTAAGGACCCTGTCATGCCC | CTACCTTGACAGGAGGCTGC | CAAGAGCCTGTGAAATGGGT | GCAAGGCTATCTCCTTTCCC |
| CSNK1E | GCAAGAGTGAGCCATGGAG | TTGAGTGGTTTATTTTTGCCTTT | CTCCGCGAATCCTCCGGCATCC | TCTGGCTCCTGTAGCCTG |
| CYP2D6 | GAGCCCATTTGGTAGTGAGG | TGGGGACTAGGTACCCCATT | CAGAGGAGCCCATTTGGTAG | TTAGAGCCTCTGGCTAGGGA |
| DDT | CTGTTTCCGTTCCTCTGCC | AGTTCACAGATGCCCTGGAT | TCTGAGGAGCTGTTTCCGTT | TCTGGAAGAAGCAGCCAGTT |
| DDX17 | CCGCTCCCAGATCTCTATCC | GACAAATCACGATGGTTGGG | GTCGTCACCAGACCGGAG | TCAACATGCAAGAAGTTGGG |
| DGCR2 | GGACGATGAACGGAGGATAA | TCTATGTACACACGCGAGCC | GGTTGCAGCCGAGAGTGT | GTGCAGGCCATCACTTCTTT |
| DGCR6 | GCGGCTAGCGGGCGTC | TTGACAGCAAGTCCCAGATG | GTCGGGATGACGTGAGCTG | CCGGTGGAGTAAGGTGTGAG |
| DIA1 | GCCACCATGGGGGCC | CGTGTGACCGTGCCC | GACAGAGCGAGCGCGG | GGGCAGGCCAGGCTG |
| dJ1014D13.C22.1 | GCTGTCTCAGATGGCCAGA | GACAGGCTGATGGCTGATCT | GGCTCCTCTTCCTTCTGGAT | GAGACGACAAATCTGGCTCC |
| dJ1014D13.C22.2 | GGGGTCATGGCTGGGC | AACTGGCTTCTCGTGCTGTT | AAGCCAGAGCCGGAGC | CTGCACAGGACAGGAAGACA |
| dJ102D24.C22.2 | GCTGCTGAAATGAGGCAAA | TACGTGAATGACGGGAACAA | GTTCTCCTGATCCTGCGTGT | TCTTTAAGGTAGCCACTCTTTATACG |
| dJ1033E15.C22.2 | ATCTCAGCGTGTGGCTGAC | CTGCAGACGCTACCAAGTGC | GTCGTGCACCCCACACTG | AAGATCTCCTCGGGGAACAC |
| dJ1039K5.3 | CCAGCCTCCTGCTCAGAC | TCTTGGATCTGCTGCTGTTG | ACTACCTACCACTGCTCCCG | CGCCTGCTTTACTTTGAAGG |
| dJ1039K5.C22.6 | ATGCGGCTGTAAAGCAGTG | GGGCTAGGCTGCTGAGTATG | CCAGATCGCGCAGTATTTCT | AAATGAACAGGCCACAGGTC |
| dJ1042K10.2 | TTGGAGCTGCAGAAGACTTG | TTGGCCTAGAGCATCCCTAA | TGAGGAGCCTTCCAGCTCTA | CTTCCTGAGCACAGACATGG |
| dJ1042K10.4 | CTTGTGTTGCCCCAGGTACT | TTAGTGCTGCTGCTGTTCCA | TCCATAGTCTGCGGAGAAGC | AAGGGAGGCAACTATGTGCT |
| dJ1057D18.1 | ACCCTCTTTCTCTTCCCGAC | CTGCCAGAAGACACCCATTT | CCCGTCGTTACCCTCTTTC | TCACACAGCTGCTATGCTCC |
| dJ1104E15.C22.4 | GACTGCCAGGTCGGAAGTAG | TTGTAACTTGGAAGCCCACC | TGCGTCCTAAGAAAGATGGG | TTGGGTTCACATTTATTGTAACTTG |
| dJ1104E15.C22.5 | GATCCTCCAAGGGAAAGAGG | TGAAGTGACCAGGGTGATGA | TTCTACTTTGCCTCCATCCG | GCAGAGAGCACAGTGATTGG |
| dJ1119A7.C22.3 | TGACCCAGATTGCAGTGAGA | CCACAGCTTAGGAAGGGACA | GGCAAATGAGCTCTGAGGAC | TGAGTGGTCTTTGGCAATCA |
| dJ1163J1.C22.4 | CGAAGTTGGGCGACTGG | CTGGAGCTGAGGCTCTTCCT | CTGCAGCTGGCGAAGTTG | ACTCTGAGGTGGGAAACCCT |
| dJ1170K4.C22.1 | GACTACAGCTCCCGGCCT | GGAGAGGGTGCTTCTTCCTT | AGGAGGCGCGGACTACAG | GCAGGAGTAAGTCACAGCCC |
| dJ1170K4.C22.2 | GGACAAACAGAGGCTCCTGA | CAGACATCAGGGACGAGACA | ACACCCTGGTCATGGCTCTA | ATTAGGCAGCAGTGGAGGAA |
| dJ1198O21.C22.1 | GTCCAGGATTGGAGGTTGAA | GAGTGGAGGTAAAGGCCACA | GCTTTCCTCAGCAGAAAGGA | AAAGGGCTGTGCTGAATCTG |
| dJ127B20.C22.3 | AAGTGAGCGCAAAGTGCTG | CAGCTCTTCCTCCCTCCTCT | GGGGAGGGGGCTCGTGTC | TACCCCCTGTGAATTTGGAG |
| dJ130H16.C22.1 | GGCCTGGGTCTAGTTGGC | AAGTCTTTGGAAGCCTGGGT | GGCTCCTTTAAGGCTCGG | GTACACAGGTGTCCGCGTAA |
| dJ130H16.C22.2 | GCTGGGATGGCGAAGAG | AGTTATATGGAGACCCCGCC | GAGGTGGCCGCTGGAG | CAGCCTGAGTGCCACTGTAA |
| dJ149A16.6 | GCTCTTCAAAGCGGAGCC | TCCACTTCAGAGAGGGTTGG | CTCTGAGAAGCCGGACTACG | TGGAACAACCAAGGAGAAGG |
| dJ149A16.C22.10 | CCAGCACAGCAGAAGATCAG | TACTTCCTGGGGTGAATTGC | GGCAGAGAGAATCGAAGGTG | TGCCTTATTCTGGGTTCCTG |
| dJ151B14.C22.4 | CAGACATGGGCCAAGGAG | GATGACTTTGGTGGAAGGGA | TGGGCCAGCAACAAGTTAGT | GACATCAGTGTCTGAGGGCA |
| dJ172B20.C22.3 | TTCCATTGCTTTCATCCTCC | CGTAAGTCTTGGGGAAGTGG | GGGAGAGGAAGTTTGGTCGT | TTGCTAATAAACCTGACAAGGAAA |
| dJ186O1.1 | GTGAGGGGCGGAGCTGG | GAGCATCACCATTTCTTCAGTT | GCTTTCTTCCCGAGGGC | GCAGTGCTTAGTGGACAGCA |
| dJ186O1.2 | TGTGGCCAGACAAGAGGTC | TGGTTTCCTGACTCAAAGGG | AACCAGGTGGTCGAAGGG | TCTTGCTCTGTGGCTGCTAA |
| dJ215F16.1 | AGGAAGTACGCCACTCTCCA | TCTGCTACCTGCCTTCACCT | TTCCAGCTCTTCTGCTGTGA | ACTGGCATGTTCAGGGTAGC |
| dJ222E13.C22.1 | AGAGCGATGAGTGAGAACGC | GACTAGTGAGGCCTGTCCCC | GACGAGAGCGATGAGTGAGA | CAGACTCTGTCTCCCCCTTG |
| dJ222E13.C22.3 | GACTGCTTTCGGCTTGCTC | GAAACAGAGCCACCCTCCTC | GTAGTTCGTCGCTCCCTAGC | CACAGGGCAGAACACAACAC |
| dJ345P10.C22.4 | AGCTAGGCGGAGAATCGAG | ATTAGCCTTGAAACAGGCCC | AGGCTCATTCTCCAGTCAGC | GAAAATTCATGAAACCCCCA |
| dJ347H13.2 | AGAGCCCACCAGGCTCTC | GCCAGCTTGCACAGTAGTCA | TCCACGAGCCAGAGAGAGAC | AGGAGAGAGGCAGTGGACAG |
| dJ347H13.4 | GTCGGGTACGGGAAGGTC | AGCATTGTCTACATGCGGC | CACAGTAGTCGTTGCCGGT | AGGACGCTTAGGAAACAGCA |
| dJ347H13.5 | GGTCGCCTAGCAACAGAAAG | ATCTCCTTAGCATCGGCCTC | CAATGCGGGTAAAGCACAG | GAGGAGCAGGGCCTAGATG |
| dJ353E16.C22.2 | TAGTCAAGCACCCAGCTTCA | GCAGATGGGTCATGCTTCTAA | GGAAGTGGGTTGAGTCCTGA | AAAGGTGGGTCAGTCCCTCT |
| dJ355C18.1 | ACTGGTGACACGTGGCTGTA | GCAGTAGCTCAGGGCGATTA | TCTTGCTGGAAGTCCCTCAC | TGCTCCTGCTGTTACGACAC |
| dJ366L4.C22.1 | GTCGCAGCCTCCTCGTCT | CCCAAATCACAGGTGGAGAT | GGTTTTGGCAGTAGCTGTGG | TTCCCTTGAGACAAACGTCC |
| dJ366L4.C22.2 | GAGTGACCACGGCTAGATAGG | TCCACAATTAAAGGGGAATCTT | GCTTTTCCCCACGAGTGAC | ACTTTAAACTATCCACAATTAAAGGGG |
| dJ370M22.C22.4 | CGGGGCGCCATGGCCGAGTC | CGTCTGATGTGCAAACTGGT | TCCAGGTGCGGCTGTGGGAC | TGTGACTGAGATGCTTCGGA |
| dJ37E16.4 | ACTGTTGAGAGGCAGGAGGA | CGTGTGTGCGTATGTGTCTG | ATACCAGCTCAGGAGCCCTT | TCCTGAAGAAGGTGCAAGGT |
| dJ37E16.C22.6 | GCTGCATGGCGCGCTGCGAGAG | CAAGGGCCCTCAAACCTT | CGGCCGGCTGCATGGCGCGC | CAGCAAAGAGTGCCCAGC |
| dJ37E16.C22.7 | GGCGGCCTCACTGCTA | AGACGTCCCAGCAGAAACAG | GAGTACGCTACACCCGGAAG | GACCTTGTCACTGGAGAGGG |
| dJ388M5.C22.4 | GGAGTGACCCTTCCTCGTC | AGAGCAGGAATCAAGGGACA | CCCTCCAGAGCCCACATT | TTCTCTCCCTGATGAGACGG |
| dJ402G11.C22.4 | ACAGGTGCAGGAAGCCG | AATCCTTTATTTGGGTGGGC | CCCACAGGTGCAGGAAGC | ACAGGAAGGCACAGTGAGGT |
| dJ402G11.C22.5 | CACCAATTCGGCCAGTTC | CTGACTGGATGTCTCTGCCA | GCTGTTCTTCAGCGGCAAC | CAGACAGAGACGGGTCAGGT |
| dJ402G11.C22.6 | CTCTCACTCCCTGTCTTCCG | ACCTTTATTGTGCACGTCCC | GCCTGTCATTCAGCCTCTTC | CCGAGAACACCTTTATTGTGC |
| dJ402G11.C22.7 | GTGGCAGGCACCACTAGC | CATTTCTGCGGTGTAAATGC | GAGCCTGCCTAGGTTCTGTG | GGTGTCATTTCTGCGGTGTA |
| dJ402G11.C22.8 | GAGGGCCATGCTGAGCCTCG | ATATGCCAAAGTCAGGGCAG | GGCAGCCTAGGCCGGGCGAG | GGGGTCACTCGAGGGTTTAT |
| dJ402G11.C22.9 | CGCTGTTTGAGCACAAGTTC | CCACACGACACAGGCAGA | GGATTGGCTGTGAGGACAAA | CATGCGGACAGAGGTTGG |
| dJ408N23.C22.2 | ATACCCAGCACACCCACTTC | AGCACCAACTGTCACCACAA | TATCACCACCACCAGCAGAG | TCTTTCTACCAGCTTCCCGA |
| dJ430N8.C22.1 | ACGAATTCTTGTTTGGTGCC | CAACCCATGAATGAAGTCCC | CTTGGAGGCTGTCCAGTAGC | TCCCGTGTAAGTCAAACCAA |
| dJ439F8.C22.1 | CGTCATGTTAGGGTGAAGCA | GAACCACAGGTCCAGGAGAA | GTTCCCAGGAGCTGATTGAT | TCCTGTGTGTCTTCTCCACG |
| dJ466N1.C22.4 | AGGGAAGTAGGTCCGTTGGT | TCCTCACCAGACAAGTGCAG | AAGGGTAGTCATTGGTTCGC | AGATGCTGGAAACTGTGGCT |
| dJ494G10.1 | GGAGGTCACTTTAGGGAGGG | GATCAGCTGGAGATGGTGGT | CAGAGCGGCCTGTCTTTATC | CATTGCTTTGCTGGTGTCTG |
| dJ508I15.2 | TTTCTCCCAGTGACAAAGCA | GCACGTCACAGAGGACTGAA | GGGAAACGGAAACAAGGTCT | AAGGGCAGACCCACTGTAGA |
| dJ508I15.4 | GAGTCCACATCCCACCTCAT | CTGTTCACCCACTCCCAGAT | CTCCTTCAGCAGGACAGAGG | CTAGAAGTCAGAGCGCCGAG |
| dJ508I15.C22.5 | CAGTCATGGCTGCCGCCGTC | CCATGAGACAATGTGGGGAT | GGTGTCCCCTACAGTCATGG | TAGTGTGGAGGGCACAGTTG |
| dJ526I14.C22.2 | GCCATGGAGGCCGAG | CCTGCCCCAAGCACAG | CGGGTGCTGGCG | CAGGGCCGGTGT |
| dJ526I14.C22.3 | GAGCATGGGCGCG | CGGCTTCCCTGAAGGGC | GCACGCCCGCACGC | GGGCTCGGTCTCCATGG |
| dJ549K18.C22.1 | CATGTACGACGCAGAGCG | ACACAGCAATGCGGAGGTAG | ATCCCGACCCAGATCCTAAC | CCATTAATAGGGCCACGAAA |
| dJ569D19.1 | ACCACAGACTCTGGGAGGC | TCCAGTCTGGTGCCTAAGGT | ACCTGGCTCAGCAGGAGG | TGAGAAGGCAGACGAATGTG |
| dJ579N16.3 | CTGGCTGAGGACACACTCG | TCCAGGTCTTTATTGACGCC | GGAGGGTGGATGGTCATTTA | GAAGCACAACATCCTTCCAG |
| dJ579N16.C22.4 | CCTGTTATGGGTCAGCCTCT | GTCCCTTCATGACCTCTGGA | CCGACCTGTGGTCTGGAA | TCCTGAACTGCTGAGTTCCC |
| dJ63G5.3 | GCCATGCTGCGCCTGGGGCTG | GTCGGATGTTGGTTTGTTCA | GCCAGCGCCATGCTGCGCCTG | CCTGTCCGTTATTGTCGGAT |
| dJ671O14.C22.2 | TGCACTCAGTAGGCCTTTGTT | CACGAATGCACAGGAAACAG | CCTTCTCGAACCCTGCTATG | AATCCAACGATGGAGACAGG |
| dJ694E4.C22.2 | GACTCCATAATCTGCATCTGTCC | AGGACCCTCCTTCACCAGTT | GCTCCGTAGGCTCAGGTAGA | CTGTGGATGGCAGAGACTGA |
| dJ742C19.2 | TCTGCTAAGGAAGCTGTGGC | CCCAGGGAAAGTCATCTTGA | TAGTGCCTCAGACAAGCAGG | TGGCACTGTGGAGCTTAGTG |
| dJ756G23.3 | GCTTGGCGAAACTGAGGTCT | ACCGGGCAGAATTTACACAG | CAATATGGCTTCCTGCACCT | TTCCAGCTTTGGGAACTCTG |
| dJ796I17.C22.2 | GGAGAGGGAACCATGGGGAC | GACGGGGTTTCCCACAGGG | CTTCTGCCCTCAGCAGCA | ATTTATTGACGGGGTTTCCC |
| dJ821D11.1 | GAGGGCAAGCGAGGAGA | CCCAACAATTATGCCCTTTG | GAAAGAGTGCCGCCTCAG | GCTTCAGCTGTCATCCATTTC |
| dJ821D11.3 | CTGTTGGACCTTCGAGCCTA | ACAACCACACCTCCACCTCT | GCTTTCAGTTGCTTTGCTGTT | TGCACAGGGTCATATTTCCA |
| dJ858B16.1 | GCGGGAGTTAAGAAGCAGGT | TGTTCTGTTCCTGGAGGTGG | AAACCGATTCTCATTGCTGC | CAGTAATTCATTTAATTGCAAAGCA |
| dJ889J22B.1 | GGACCCTGAGGACACGG | GGGAGAAGGTGCAGGTATCA | GAGGCGACTGTAGCGTGC | GTCCAAGGGTCTAGGAAGGC |
| dJ90G24.C22.6 | TCCTTGTGCCTCTTGGACTT | TTGGAACACTAAAGCCGGAG | GAGGAGGAGCTCCGACTACA | AATGATTTGAGCTGGGCGT |
| dJ930L11.1 | CGTCAAGAAGGAGGTGAAGC | CTGAACTCCACGCTTGTGAA | GCTCTCGTTTCTTGTCCCAC | CCCATCTGCCCAGAAGTAAA |
| dJ979N1.C22.2 | GCATACCATTTATCGGGCTC | CTGCCCTGACCCTTCTATCA | CCGCCCGTAATTAAATAGCA | CTCCTCAACCAGGAGAGCC |
| DMC1 | GGAGACTGTGGGTACGAGGG | CAATTTGCATCAATTCACCA | GGCCCGAGTTAGAATCCTGT | GATGTGAAATTGGAGACTGCTTT |
| DNAL4 | CCTGAATCTCCTGGGTGTGT | TGGCTCAGGAAACTGGTACA | TCTTGTTTCGCTCCTTGACA | GTCAAGTTCACACACTGGGC |
| DRG1 | TGTGAAGGGAGACAGTGTGG | GTAGGGTGCTTGATCATGGG | AACTCTCTCGCGGTAATTCG | ATCCTGATCCAGTGACTGCC |
| ECGF1 | CGGACGTGTCAGGCATC | AAAGGAGCTTTATTGCTGCG | GACACCGGAGAGACACGG | GCGGCAAAGGAGCTTTATT |
| EIF3S7 | CAAGAGTGCTGCTGCTAACG | CTCGTAGTCTCGGTGGAAGG | GCTTTACGGCTCGTGAGTTC | TTCCACTAAGCATCAAAGGC |
| Em:AC000068.C22.3 | CCCAGACATGGCGGAC | TGCAACAGCAACTTGAAACA | CCGGTCGCCCAGACAT | TCTCCTATGCTACCATGCCC |
| Em:AC002073.C22.2 | TCAATCACCTGCAAGACGAA | TTAGGACCCTACCCAGCCTT | AACAGCATAACAAGGGCAGG | GCAATGTTTGGAAGCCCTTA |
| Em:AC002378.C22.1 | ACGAGCTGAGGTTGGACTTG | ATTGACCCGCTTTCCTTATG | CTATTCATGAGCCGAGGAGC | GGTGCCTCATCTCTAACCCA |
| Em:AC002472.C22.7 | CCATCTGTGCTCCTGCCT | CTTTGCCTGCCCAAGTCTAC | GGCTCCAGCGTCTCTAAGG | GAGGGTTGAGCATCTTCTGG |
| Em:AC002472.C22.8 | CTTAACTCCCGCCTCTTTCC | TCCGCTTTATTATGGCACCT | AAGTCAAGAGAATCGGCTGG | CTCCGTCTAGAATCCGCTTT |
| Em:AC004471.C22.1 | TGGGATAGCGATGGAG | GGAGTCCTCTGCTGGGTGTA | TTTCCTGGGATAGCGATGG | GGGCCAATTAAACAGCAAAC |
| Em:AC004832.C22.1 | ACTGCAGTGGAGCCAGTACC | GCTTCTGTCCAGCTCAAACC | ATTTCCTGACCTGTCCTTCG | GGTCCACTTCCACTCAGCAT |
| Em:AC004832.C22.5 | GACCTGCAGAAATCCGAAGA | GTGCACAGGTAGAGGTGGCT | TGACTACTTCCTCCTGCGCT | AATGGTGACGTGGGACAAGT |
| Em:AC004882.C22.1 | GTGTGTCTGATCTGCTGGGA | CTCATCGTCGTCATCCTCCT | AGGCCACCTCGTGTATGC | GGCTTCTGCCTTCTCTCTCA |
| Em:AC004997.C22.11 | TGAGCTGTCCAAGGTCACAC | TAGTCACAACAGCCAAACGC | GTGAATCTCTGACAAGGCCC | CTGCATAGACAGGGACGTGA |
| Em:AC004997.C22.8 | GCCAGCTCGCCGGGTC | GGGCAGCTAATCATCGGAGT | CCACTGTTGGGCCAGCTC | CGTCACAGGATTGCAGAGAA |
| Em:AC004997.C22.9 | CAGACACCTGCACAGAGGAT | TCTCAGCCTGACCAAGACCT | CCCATAGGCTCGTCTGAGAA | TATTACAGTGGGTGGGCTGC |
| Em:AC005003.C22.4 | AGCGCACTCGCAGGTC | CTCTAGCGTGGTAGCATCCC | CAGCAAACCAGCTCCCAC | TGGAACACATACTTCGCTGC |
| Em:AC005004.C22.1 | GCTTGGAACAGCTAAAGGGA | CCCACTCACTGATAGCAGCA | GCAAGATGACTTCTCTGCCC | CTCTTCCCAGACACTGGAGC |
| Em:AC005005.C22.5 | GGGCTTTGTCTGACTGGACT | GGTTACCCAGAACAGCAGGA | TTACAAGCCCAAGATCACCC | TGAGCTTCCTGAACACCACA |
| Em:AC005005.C22.6 | AAACTGATTGACTGGGCTGG | CAAGATTAGCCAGGAAGCCA | GTCGGAGGTCTTACCCAACA | CTGGTCTCGAGTTCCAAAGC |
| Em:AC005006.C22.2 | GCTCACTGGAACTCAGGCTC | GTCTGAGGTGGCACCAGG | CGAGGGTCTCACTGTGTCAT | ATCTCGGTTAGGCCCTCTCT |
| Em:AC005006.C22.4 | ACCTGTCTGAAACGGGACAC | AGCCCTGTTCTCCATTCCTT | AGCATAACGTGGGCACTAGG | GACTCTGGAAGTGCTGGGAT |
| Em:AC005500.C22.3 | CTCATGGAGGGCGCAG | CCTGCTACAGGGTGGGTG | GCTTCCGGCCGCTCTC | GGTCTTTAAGCGGAACCCTC |
| Em:AC005500.C22.4 | GGCCACAGTTGTAAGGGATCT | CACAGGGAGCCTAGTCCTCA | CACCAGGCCACAGTTGTAAG | CTCACTGAGGAAGGCCAAAG |
| Em:AC005529.C22.5 | CAAAGCACCTGCTCTGAGTG | GGCTTGGCTTCCTATTGTGA | GACTACGAATCCCAGCAAGC | AGGTGGAGGAGAGTGAGGG |
| Em:AC005529.C22.9 | TCCAAGATGCCGTTCCAC | CTGAAGTACACGCAGATGGC | AGCCTCCAAGATGCCGTT | GCCCTGAAGTACACGCAGAT |
| Em:AC006547.C22.2 | CCTCTGAGCACTTCCCTTGT | TTGGCTGCCTACCTCTGAAT | ACTAAGAGTGGCTGGCGAAG | CCACCTGTCTTCCTGGTCA |
| Em:AC006547.C22.3 | GACCTGTGTCAGCAGAGCC | ACACAGATGGGTAACACGCA | AGGCTGCTTGAAGACCTCG | TCAGACTCTGCTGCTCCTCA |
| Em:AC006547.C22.4 | CAGCGGACTTGTGCATGTTA | CATGACTGCTGGTCTCCTCA | CAATGTGGCCAGCTTGACTA | CTCCACATGTGTGCTGTGTG |
| Em:AC006547.C22.5 | CTGTTCCCGTCTACAATGGC | CATGGGCAGATGAGTGGAC | GCACCCTCTTCTTCGTGTTC | GACACAGACCAGCCCAGTG |
| Em:AC006547.C22.7 | CCTTGAGGAAAGGAGAGGCT | ACAACCAAGCAGGAGTGGAC | TTTCCGTATCAACAGGAGGC | CCCATAACACCTTCTCCAGC |
| Em:AC006946.C22.1 | CGGCGACGGCCGGATGGCTG | ACCCAGCAGGGAAAAAGAGT | GTGCACGTGCGCACGGCGAC | GTAATGCCACACTCATGGGG |
| Em:AC006946.C22.2 | GGAGGACAATGCGCCC | GGGGACTCTCAACCCAAAA | GGGAGGGAACAGCTGGG | TTCTGGGTCAATTTCAAGGC |
| Em:AC007050.C22.6 | TCGTGGAGTTCTTCCCTGTT | AATGGTTCGCTGAAGTGGTT | CTATTGAAGTGCGCCACTGA | TGCAGGCAAGTTGAAGATTG |
| Em:AC007663.C22.1 | ACGACTTTCAGTCCCCGA | TGTAAACATGATGGGGTGGA | CCGAAACGACTTTCAGTCCC | TTCCACGTCGTCCGATATTT |
| Em:AC007663.C22.3 | GCGGCTAGCGGGCGTC | ACTCTCTGCCTGGTCCTGAA | GGATGTCGTGAAGCTGGG | CCGGTGGAGTAAGGTGTGAG |
| Em:AC008079.C22.1 | GTGAAGTCGTGCTGTCCTGA | CGTAGATCCAGGAACGGAAA | CGCTTGAGAGATTCCATCGT | TGGTGAAAGCATCCATTCTG |
| Em:AC008101.C22.3 | CTTGGACCCGGACTCGTTA | CTGTGGATGTGTCGCTCTGT | CGACGTCTGAGGACCTGG | TCTGTCGCTCTGCTTCTGTG |
| Em:AC008101.C22.5 | TCTGTAGAATGGGCACACGA | TTTGATCGTAACCAGGAGCC | ACCTTGGAACAAGGAGCAAA | GAGGCGAGGTGAGTCTTTGA |
| Em:AC016026.C22.2 | TGAGAGTGAGGCAGCA | AACCTAAAAGGGACTGGGGA | AGTGCTGAGAGTGAGGCAGC | TGAATCACCCAGAGTCCCTC |
| Em:AP000344.C22.2 | ACCACTCACCCTACCTGCAC | TTCGGCAAGTGACTAAAGGG | ATAATGTGCTCAACCCTGGC | GGTTCAAGAAGGAGGACACG |
| Em:AP000346.C22.6 | GAGTGGAGAGGCATGGAGAG | TCTCTGCTGGGACCAAGAGT | CAAAGAAGACAGCAGGTCCC | ACTGGGTCCTGGATGGTACA |
| Em:AP000347.C22.3 | CTTCCCAGCTCTCCCTGTC | CTGGTGTCTACGGTCTGGGT | TAATTCCCACGAGAAGGCTG | AGGAGCAGTTGGTGATGAGG |
| Em:AP000348.C22.3 | GTCTCTGCTCCACGCTTTTC | AGCCTGTTGTGTGCCTCTCT | TCTTGGACTAGCTGCAGGGT | CACAGCCTGTTGTGTGCC |
| Em:AP000348.C22.4 | GCCGCCGTCTCTAAGGTC | GAGTCTGCACCGACCTCTTC | GCTGCCGCCGTCTCTAAG | TGCACATTTGTGTCTTGGGT |
| Em:AP000350.C22.1 | GCGTGCTTGAGAAGGTTCA | TCTCCTTCCAGACCCAGAGA | GTTAAAGAGCGCGTTGCTG | CTCTGCCTCAGATTCCCAAG |
| Em:AP000350.C22.2 | TGGGACAGCAAGACCTCC | AGGAAGCAGAGGACAGGACA | ACCGCTTGCTAATGGCAG | CGCTCACCATTGATTGCTAA |
| Em:AP000351.C22.3 | CGCTGTCCTTGCCGCCC | AAGCTGGAGAGAAGGGACTGG | CAGCGCCTTCACTGCCATC | TATGTATGCTGCACCTGAGGA |
| Em:AP000351.C22.5 | CAACATGGCCCTGGAGCTC | CTCAGATTGCTAAGCCAGCC | CTGGCATGTGTCTCAACATG | AAGGAAGGTGGCTCAGATTG |
| Em:AP000352.C22.1 | CAACCTTTGCCAGTGATGAG | GACCTGGGTACAGGGAGGAT | CTGGAGAGTTGTGGACTGGG | CTGGGTGTGGGCAGATTTAC |
| Em:AP000354.C22.2 | ATGCATCACGAAGAGGCAG | GTAGCTAATGGCGTCGGTGT | CAGCCCAAACCAGGAGATT | TTGCTGGAAGCTTTACAGGG |
| Em:AP000355.C22.2 | GCTGTCTGGGAGCGAGAGTA | AGCCTGCCACACTTGCTAAT | AGTGGCGTGTCCTCACTTG | AACCTGGACAATGTTATTGGG |
| Em:AP000356.C22.6 | GAAGCTGTAGCCCGCTCTG | ACATGACAACACACGGCACT | AGAAGGAACTCGACACGCAC | GCATCCGTCAAGCAACTTCT |
| Em:AP000356.C22.8 | TTGAGAGGACCCTCCTTCCT | CCTTCATCGCCCACTATCAT | CATCCATGCATCACCAGTTC | TGGCCACCTATGAGTCCATT |
| Em:AP000546.C22.2 | AGTCTGCTGGCAGGAATTGT | CAGCAGGCAGATGATGACAG | TCTTCTCCTCGGGTGGTATG | AAAGCCCTGTACTGAAATCACA |
| Em:AP000553.C22.3 | CCCTGAGCTGAGTGAGGAGT | CTTTGAGGTCAGAGGGCAAG | GTCCCAGCTGTGTGGACAGT | CAGATCCGAGGGACACCTTA |
| Em:AP000553.C22.6 | GCCATGTCCCGCCCTC | TAAATCCTGGCTCCCCTTTC | CTGTCCGGCCCACTCC | CCTACCCAGGGAAGTCAACA |
| Em:AP000553.C22.7 | CTGAAGAGAGAGGTGGGCAG | TGTGGTGACTCCTGCTCTTG | GCAAATAAGCAAGTGGAGGC | TCACTGGCTCCCTTGAGAGT |
| Em:AP000557.C22.1 | TCTGGGTGACTGTGTTCGTC | CATCACCTCGCTTCTCCTTC | CTCCCTAGACCTGTTCGCTG | TCCATTAAAGCTCTGGGTGTG |
| Em:AP000557.C22.2 | TGAGGTGTTTAGTAGGGGCG | AGGAGAGGAGTGCACCAGAA | CAAGGAGTACTTGAAGCCCG | GGCAACCTTGGTCTACACTCTC |
| Em:AP000557.C22.3 | ACCTGTAGTTCATGCGGGAG | ACCATCCACTGATTGTCGCT | TGTGTTTCACAGAGGAGCTAACA | TGTCCAGTGCAAACAGAGGA |
| Em:AP000557.C22.4 | TCCAAGTGGGAGGCACAG | GTGTGAGGGTCTGAACGCTT | GCAGCTGGCTGACCGTCT | GCTTCTCCGTATACCTGCCA |
| Em:D86995.C22.1 | GAGACCCTCTTGCCCTGG | TGAGGCTTCTGAGGGAGAGA | CGAGGAGACAGCTGAGGC | CTAAGCTGCCTTCCACCATC |
| Em:U51561.C22.2 | GAGTTGGAACCTCCCACTGA | CTTCAAGCCGGTAAACCTCA | AATCTGAAGCCCACCACATC | CTGGGGAAGCTCATAGACCA |
| Em:U62317.C22.13 | CGCTATGGGCTCGGAC | ATGCGGACTCGCAGACTTTA | GGAGCTACCACAACAGGTGC | CGATGCGGACTCGCAG |
| Em:U62317.C22.14 | CAAGAAGCTCCGAGACCAAC | TGTCCAGAAACAAGAGCAGG | ACTCTGAGGACAGACACGGC | AGGAAACAGTCTGGGTGTGG |
| Em:U62317.C22.2 | GTACCCCTCGGAAGGCAG | ACACGGCTCCTCAGTACACA | AGAACTAGTGGCGGGCTGAG | TGAGATGGCAACACTGCTTT |
| Em:U62317.C22.9 | GGGCCGCGTAGCGGACATGG | ATAGTGGGAGTCCTGGGGAG | TGCTCTAGCGGGCCGCGTAG | TAGACTCAGACCCCCACAGC |
| EP300 | TTTCCTCGCTTGTATCTCCG | CCCTCAGGTTCATCTTGCAT | TTTCTATCGAGTCCGCATCC | TGAACCACACACACAAAGGG |
| EWSR1 | GGACGTTGAGAGAACGAGGA | AAACTCCGCACACTACCATTT | GCCTAGAGGGAAAGCGAGA | CCTCTTGGTTCTCCACAATGA |
| FBLN1 | GGCGGGATAATTGAACGG | ACTGGCAGCAATGATTTGG | GGGAGGGAGGACCAGGAG | AGTTGAGCAACTGGGCTCAT |
| FBXO7 | CTCAGCTCCGGTAGTCGC | AACACTCGAGATCAGCACCC | TATTCCAGAGACCGAGTGGC | ACTATTCCCAAGGCCAACCT |
| fF1A6.C22.2 | CAGCGCTACCCGCCAT | GTCCGGCACATTACAGGTCT | CTCCGGCTGCGTCTTC | CACGGGACATCCTTCTGAAT |
| G22P1 | CCAAAGTGAGCAGTAGCCAA | TAACTGGCTGAGGACAAGGC | CTGTCCAAGTTGGTCGCTTC | GAGGGCTACACCATCACCAT |
| GALR3 | GAGATGGCTGATGCCCAG | CAGGGTTTATTCCGGTCCTC | GTCTGATGGGGAGATGGCT | AGGCGGCAGGGTTTATTC |
| GAR22 | GCGGGATGAGCCAGTGA | CAGCTGTGTTGTTGGTTTGG | ATCCGAATTCCAGGGAGG | AGGAAGCAAGAGCTGGACTG |
| GCAT | GAGGTAGGAGCGATGTGGC | AGCTCTGGGACTTTGGTTCA | GGCGAGGTAGGAGCGATG | TCACATTCTCACAGCCCTCA |
| GGA1 | CTCGTTGGCCTCTCTATGGT | AACAAAGGGTGTCCTTGGC | TGCCCTTAGCCTGCAGTATC | CTCCTACTGGGTTTGGCTCA |
| GGT1 | TTCCACCAAATCCTCCTGTC | GGCACAGTGGCCTCATTTAT | CTGCCTGATCAGAGAGTCCC | CCTGGAGCCTGGCACAGT |
| GGTLA1 | TCTGTCGATCCATCTTCGTG | CTGCCAGAGTAGTTGGTCCC | TGTAAGCCCATCTCTGTCCC | TCAGCCTCTCATCTGCCC |
| GNAZ | TTGTCTGCCTGGTCTCAGTG | GTGGGTTTCACCATAGGCAG | CCGAGGACAGGGAATGACTA | GGATTGGGCCTCTCTAGCAC |
| GNB1L | ATTCGCGTAGCCTCAGGTAA | GTGATGAGGCACTCCACAAA | GCGATGTAATCGGTCTCTGG | ATACCCTCAGCTGGCAACAC |
| GP1BB | CTCCCGCTGCAGAGTAAGC | AGGGTCCTGTCGAGTTTGC | GCCTCTGGGCTATTTCTGG | GTAGGGTGGGCAGGAGTTCT |
| GPR24 | AGCCTGGGACTGAAGAGGTT | GTGTTGTGGTGCCCTGACTT | ATTCAGAAGTGGAAGCCAGC | CAAAGGTCTCATCCTGCTCC |
| GRAP2 | AACTCGGTGTCAAAGCCAAG | CTCTTTCTTGTGGGCAGCTC | TGGAAGACAGCACAAAGTGG | CAGCCCAATTGCCAATAAAT |
| GSTT1 | GTCGGTCGGTCCCCACTA | GTGAGGTGCTCTGGGACTTG | ACTCCCTCTGGTTTCCGGT | AGGCTGAGCCCAGGTTTATT |
| GSTT2 | GCTGTCCTTGCCGCCC | AGCTGGAGAGAAGGGACTGG | AGCGCCTTCACTGCCATC | ATGTATGCTGCACCTGAGGA |
| GTPBP1 | CGCGATGGACTCGCCGGTCC | GCTGGGTGGCGGTCATAG | AGCGCAGTCGCTCCGCGATG | GTCTGGCTACCTTCGTCAGC |
| GTSE1 | AGGGGACGTGAACATGGA | AAGTGTAAGCCACTGCGACC | TGACTTCTGACAGCTCTCTCCA | GCCTCCCAAGTTCTAGGGTT |
| H1F0 | AGACTGGCCCGGTAGTCAG | TGGTGGTGAGCATCAAGAGA | AGACCAAGCGACAGACCG | GTTGCTGTCCTTGCACAACT |
| HIRA | CGAACAATGAAGCTCCTGAA | TCTCCTGCCAGTGTCTCCTC | GGCCGAACAATGAAGCTC | AGCACATCTCCTGCCAGTGT |
| HMG2L1 | ACACATTCTCAAAGGCCCTG | TGGCTAAACCTTTGCCATTT | AGACCTGGTCCTGTAGACGG | GTCTGGAGGTCCAACTGAGC |
| HMOX1 | CCGAGCATAAATGTGACCG | TAAGGAAGCCAGCCAAGAGA | AAGCGATCTACCCTCACAGG | TAGGCTCCTTCCTCCTTTCC |
| IL17R | CGTTCGTTCGCTGCGTC | CTCTCAAAGCTGGGATCTGG | GAGCCGACTCGAACTCCAC | AGACGATAACCAGACCGCTG |
| IL2RB | GCTCCACCCTGTGGATGTAA | ACCCTCAACAGGGTCCTTCT | ATGTCTCAGCCAGGGCTTC | TGATTAACGAGGGAGTTGGG |
| KCNJ4 | GGACTCTCGTCGGACCCT | GTGCTGGAGTCAGGAGGAAG | ATCGCGCCTTGGGATGTA | CCCAAGGTTCTGAGAGCAAA |
| KCNMB3L | TCCTTGCTTCCACTGACCTC | TTGTTCCCTTCTTGGCAATC | CTCATCCTTGGACCCACAGT | CCAAACACGGAATAAAGGCA |
| KDELR3 | TTCCTAGAAGTTTGCTGGGC | TTCATGTGCTTGTTAAGGCG | GGGCACCTAGAGACCGGG | GAAGGTCTTCTGGGTGGGAT |
| LARGE | GGGATTAGGGATTGCCACTT | GCTAACCTCTGGGAATGCAG | CACGGCCAAGAACCTACATT | GACTCATCTAGGTGGGCTGC |
| LGALS1 | AGTTAAAAGGGTGGGAGCGT | AGCTGCCTTTATTGGGGG | TGACTTGCAATTGGCTGAAC | CTGGTTCAGAGGGAGCAGAG |
| LGALS2 | CGGGGAGACACAAGGTAGAA | GTCTTTTATTCTTTTAAC | TCTTCTGCCTGTTTGTGCTG | TCGGCTGGAAGTCTTTTATTC |
| LIF | CTCTGAAGTGCAGCCCATAA | CACATCTGGACCCAACTCCT | CTGAGGTTTCCTCCAAGGC | GTCCACAATCTCCCAGAGGA |
| LIMK2 | GCCTCCTCCTCCCCATTT | CAGGGCTAGGGAGGTGAGT | GGAGTTGTAGGGAACTGAGGG | GCAATGCTGGCTGTAGAACA |
| LZTR1 | GGAAATGTGGTTTCTCCAGC | CTTCTCAGTAGGCAGGGCAG | GCTAGGCTTGTCGGGAAGAG | TGGGACCTGTGTCTTCAGTG |
| MAFF | AGAGGGCACCTTCTGCAAAC | CTGCAGAGAAATGGGACCTG | GGGAAGCTCGCCTTACAACT | AATGTGTGTGCACCAAGGG |
| MAPK1 | CGACAAGAGCTGAGCGG | AACGGCTCAAAGGAGTCAAA | CTGTCGGCTCTTCAGCTCTC | TACATACTGCCGCAGGTCAC |
| MAPK11 | ACATGTCGGGCCCTCGC | CAGCACCTCACTGCTCAATC | GGACATGTCGGGCCCTC | GGCCAGAAGTCTGTGACCAT |
| MAPK12 | CAGGCTCTGCGGGGTG | GAAGGTGAAGGTGGTCCTCA | GTCGGCACCTGGGACATC | GTCAAGGTGGCAACGAGAGT |
| MAPK8IP2 | ATCGAGGCTGACCTGAGAAG | ATGGTGGCCTCTTGAGACAT | GAGACAGCGCTATGCTCACC | CAAGCGTAAGGTCCCATGTC |
| MB | CACCCAGTGAGCCCATACTT | TGCAAAGCCAACTTCAACAC | AGCCATTGAGCGATCTTTGT | GGAAGAAGTTCGGTTGGGAT |
| MCM5 | GGTTTGTGAAGTGCGGAAA | CGAGTCCATGAGTCCAGTGA | CTGAGCGTGGAGGTTCTTGT | GAGGTCCCAGCAACATTGTC |
| MFNG | CTCCCTGTCTGGTTGGGATT | CCAATTGCCACTCAGATCCT | CTACCTCTTCCCTCCTTGCC | ATTCAGCCTCCTGAGGGAGT |
| MGAT3 | TCCTGTCTCTCTCTCTCCCG | GCCCTGTCACAAACCCTATC | GGCGATGGGATGAAGATG | GTCTTGGCAGTCATAAGGGC |
| MIF | CTCCTGGTCCTTCTGCCAT | GTCCCTGCGGCTCTTAGG | CAGTGGTGTCCGAGAAGTCA | CTAGAACACAGCGTGCGG |
| MIL1 | GATACCGTTCCGGATGTCAG | GAGGTTCAAATTCCAGCCAA | AGGGTTTGGGTCTTCAGGTC | GCCCTTGGGATTTACAGTCA |
| MKL1 | GGAAGAACTGGTGAGCCAAG | TTGACAGCTGCTCTCCTCTG | AGTGAGCGGAAGAATGTGCT | TTAACCTGTCTCAGCCCTGG |
| MMP11 | GCCCCGGGGCGGATGGCTCC | CATCCCCCTGAGGAGACAT | AGCAGCCCCGGGGCGGATG | GGTTGTACCCCACCCCAT |
| MN1 | GGAGAGGAGCTAATACCCCG | AGAGGAAGGGCCTGGTAGAG | CTCGCAAGATGTGAGAGGC | TCAGCAATAGTGGCCCTTTC |
| MPST | CAGCCCGAGTGTCGCC | AGCCAACAAGTCAACGGAAT | TGCTTCCCTTCTGACATCCT | AAACAGAAAGGAGGCGGG |
| MSE55 | CATTTGGGGACCTCACCTTA | GCAACATGACTTCTCCCCTC | AGAGACGAGCCATGAACCC | CTGGCACAAAGGACACACTG |
| MTMR3 | AGCCTTGTTGGACACTGAAGA | CACGCTCCCATTCCAAAGT | CCGACTTCCTTGTGAAACCT | GTGCATCCCAAATCTGTCAC |
| MYH9 | AGCGCTCGAGAAAGTCCTCT | CTGTCCATCCATCTCAGGCT | GAAGGCTAAGCAAGGCTGAC | AGGAGGAGGCATGTTCACAG |
| MYO18B | TGTGCTGCGTGTGTCTGTAA | CTTCAAGGAGGCTGAACTGG | CGTGGTTCCTGTGATCTGTG | GATGCCCAGGAGTCAACACT |
| NAGA | GCTGATACACGCAGACCAGA | CAAGAGGGTTTACGCTTGGA | TTCTTAGCTTCCAGAGCCCA | TTCCTGGCAAGAGGTCAGAT |
| NCF4 | GCTGGAGGAAGTGAGAGGTG | AAAGACAGGAAGTCTGCCCA | AGTGGATTCCTGCAAACCTG | CCACGGGTCCATTAGCCT |
| NDUFA6 | CGAGAGCCTCAGAGTCATCC | TACCAAGGTCCCACTTGCTC | CCAGAGAGAGGCTTCCCAG | TTTTTGAACAGATGGCCTCC |
| NEFH | AGGCCATGATGAGCTTCG | ATGTTCCTTTCTCCCTGCCT | CTCGCGCACCTGCTCA | GTGTTTACGTGTGGCATTCG |
| NF2 | GGGCTAAAGGGCTCAGAGTG | GTAGCAGGAGAAGTGGCAGG | GAGGCCTGTGCAGCAACT | AGCTCCCTATGGATGGCTCT |
| NHP2L1 | GTCCGGCAAGAGACTACCAA | AAAGGATGAAGGATGGCAGA | CCTGGGACCAAGACAAATTC | CCTGCTTCTGTCTGCTCTGA |
| NIPSNAP1 | GGCCTTCCTGCAACCTTT | CTTGTCAGCCTTCAGTTCCC | TTACCGAGGTGGATCCTGAG | TGGGCAGAGCTGTAATCCTC |
| NLVCF | TAGCCATGACGGCCTCC | CATTCCTACTCTCAGGGGCA | CGAGGGTAGCCATGACGG | ACAGCATGCCTGATTCTGTG |
| NPTXR | CCTCACGCTGAAGTTCCTG | CCCTCAAGTCCCCAAAGTG | CCGCCTCACGCTGAAGTT | CTGGAGGTGTGGGTACAGGT |
| NUP50 | GAGGAGGTTCGAAAACATGG | GCAGCCGACTTTGCGTGTTC | GTTTGAGTCTCTGGGCTTGC | CAGCAGCAACTTGGCAATAA |
| OSBP2 | CCGGCTCTATGGGGAAAG | GTATTTGTTGCAAGGGTGGC | GCACGTGACTGCGCCC | GTAGGGCCCAGGTCCATAAT |
| OSM | CACGGGCACCCAGCAT | TCTCATCCACAGAGCACCTG | GAGCACGGGCACCCAG | TCTGGTTTGGGACATGATGA |
| P2RXL1 | TGACTCATGTGCCCGC | ACACAGCCGACTTCTATGGC | CTGCCATGCTGACTCATGT | CCTCTGCTAGGAACACTGGG |
| PACSIN2 | GAAAAAATGTCTGTCACATATGATG | GGAACCATCATCTCTTGCAGG | GCAGCCTGAACGGAGTGT | AGGAACACCATGAAGCCAAG |
| PARVB | CACGCGCGGCCCATGTCC | GGGATGACAGTCAACACAGC | CGGCTCCACACGCGCTGC | CAACAACCAAAAGAGGCAGG |
| PCQAP | CCAAGCGGGATACGGG | TGTACAGGTGTGGGATTTCG | GCTCTGTGACTGAGGCGG | AGCACACACGAGACGCTCTA |
| PDGFB | GAGTCGGCATGAATCGCT | GACGGACGAGGGAAACAATA | CCGGAGTCGGCATGAATC | AAGATGGCGATGGAGTTCAG |
| PES1 | CTAGTCGGCTCCTCAACGTG | CTCTTCTCTGACCAGGCACC | GAAGTGGAGCTCCCTGTACG | AATCACGGGTCCAACTGTGT |
| PIB5PA | AGCGGTAGAGCTGGAGCC | ACAGAGAGGCAGGTGCAGAT | GGTTGAAATGGCTGATGACA | ACAGTTGAGGTGCCAGGACT |
| PIK4CA | AGACCAGCATCCTTGGAGAA | ATTGTGGGACAGCTTTGAGG | TTTCAACACGGTCCTGTCAC | ACCATCCATTGATTGTCGCT |
| PISD | GATGGCGACGTCCGTG | AGGAACGGGATAGGTTGAGG | ACGCTGAGAAGGAGCAGACA | AAACGACAACCGAGACCAAC |
| PITPNB | GCTGTGAGAGGCGGTAGC | CTGACCCTACAGGGGACTCA | GAAGGGTGTAAGCACGCAG | GCTTGTTCCCCTCACTTGAC |
| PK1.3 | GGAGCTGTGAGGAGGAACAA | ATTGGCTGGTGTCTTTGGAG | TCCGGCAGTGGCTTACTC | CCAGGAACCACAGACAAAGG |
| PKDREJ | GGGCCGGCGCCATGAGGCCT | GCCTTGTGAAGGACATGAGT | CTCTCCCAGCTTCTCCTGG | AATGTAGGGGATGTGCCTTG |
| PLA2G6 | TTCCTCGTGTCTCCGATTCT | CTATGGACTCAGAGGTGCCTG | CTCCGGACTCCCAAGTCTC | GACAGAAAGTGCTGGAAGGC |
| PMM1 | CGCGGACCTGCAGCCA | TCCAACACCAGGACCTCTCT | CTTCTGCCGTTGCATCTTC | GAAGCCAGTGCCACTAGGAG |
| PNUTL1 | CCACCATGAGCACAGGC | GAGCGTCACTGGTCCTGC | CTCCGCCGCTTGTCGT | CCCTCGGAAACAGACACAAT |
| POLR2F | CTGAGGCGAGGGTGTCAT | ACTCCAGCTCAGTCGGTGAT | CAGGCGCAAGATAAGCTAGG | CAAGGGCAGGAAGATGACTC |
| PPARA | GCTGTCACCACAGTTCTGGA | AGGTGTGGCTGATCTGAAGG | TTGTGGCAAGACAAGCTCAG | GCCGGTTACCTACAGCTCAG |
| PPIL2 | TTCGTGCTCGCTAGTCGC | GGTGAGGCAGAGATGCAGAT | GGCTCCATGGTCTGAGTTGT | ATGGCGCAGTTCTTGTTCTT |
| PRAME | TTACTCTCAGACGTGCGTGG | TGCACATCCTGGCTTTAGTG | CGGTGTGGTGAACTCTCTGA | TTGTCTGAAACTGTGGCTGC |
| PRKCABP | TCTGGGATCTGAGCCTATCG | CTGTGCGAGGACAGAGGAG | GTGGGTTCAGGTACCAGCCT | CAGGTCCAGAGACCAAGCTC |
| PRODH | CCCTTGTCTGGCATTTGTC | TGAATCTGTGTGAGGATGGG | GAGGCTTTGAGAAGCCAGTG | CCCACACATTCGAGGAGAGT |
| PSCD4 | AGGAGCACGGGTCATCTTT | CTGTAGTGCACGTAGGGCAG | AAGCAGGAAGGAGCCTGTTA | CTAAGTGCAGAGAGCGCCTT |
| PVALB | CCACCCGAGTTGCAGG | GCAGAGAGGTGGAAGACCAG | CTTTCAGTGCAGGCTCCAG | ATGGTGTCATTAGAGGGCCA |
| RAB36 | CAGGCAGGTTCTCGTTGCTA | ACTGGAACATGGGCCTACAG | ACTCCAGGCAGGTTCTCGT | GGAGTTTGCAGTTCTGAGGC |
| RABL2B | ACCGCACTGACAATACCCTC | CCTGTATCACGGGCCTAGAA | GGTCTCTCCAGCCCTCACTC | GGAATTCTTCCACCAGTCCA |
| RAC2 | CTGTCACCACCGACACTCTC | CTAAGAAACGCCACAGGGAG | CCTCACCAGCCTCCACAC | GACCATCAACGAAGCTCTGC |
| RANBP1 | GGAGGGAAGGAGCTACGAGT | CAACCTGAATGTTGAGTTCAGTTC | ACAATGAGAGTGTCCGCCTC | TGAAGTCATCTTCAATAGGGCA |
| RANGAP1 | TACTGCAACTTTGGCCTCCT | GACTGACAGGACACATGGGA | ACTAGCCGGCTGGACATCT | GGCCAGCAGAAGACGTTAAA |
| RAYL | GCTCTTGTCCTCTGGGTACG | GATCTGCTCCAGCTCGTCAT | ATCGTCCTTCCTTGAACCCT | TTCTGTCTTCTCCGGTTGTG |
| RBM9 | AGAAAGCTGTGGAGGTGTGC | AATAGCCAGGCCTCATGAAC | AAAGAGAGATCAATTACCCACCC | AAATGCACAATCTTTGGAACAA |
| RBX1 | CAGACCGTGTGTTTCCAA | AAGAGAGAGCATCCGTTCCA | GTCGGACGACAGACCGTGT | TCAGAGGACAACCCTGCTCT |
| RFPL1 | GTGACAAAGCTGGGACACAA | TGAGTTGGATGGTGGCAATA | CCACCTCAGTTCAGAGCACA | GCATTTGGAAATTGATCCCA |
| RFPL1S | GCTTCCATCCCTCAAACTCA | TGTCTGCCTTCAGAGACCCT | TCGCTGTAACTTGCGGTCTA | AGGCCTGGAATCATGCTCTA |
| RFPL2 | AGAAGGAGCTACATGCCACG | GCGTTCCTAAGTCTACCCACC | TGGACTTTGAAGTGTGGGTG | TCAATGAGTTTGATGGTGGC |
| RFPL3 | TGGTCTTGTTCTCGGAGTGA | TGAGTTGGATGGTGGCAATA | TTCAAATCTCTGAGGACGGG | AGCATTTGGAAATTGATCCG |
| RFPL3S | CACGTCCACCTCCCAGTAGT | TCAGTGAAGCAAACACGTGAC | TTCTCTGCAGACTCCCAGGT | TGTCTGCCTTCAGAGACCCT |
| RPL3 | TTTGATGGCGTGATGTCTCA | GCAAAATCTGTTCCTGGCAT | ATATAGCGGACCCGTAAGGC | CCACCAACTGCAAAATCTGTT |
| RRP22 | GCTGTCTGTCTGCCCTCC | TGATTGTCCCAGTCACAAGG | CTCCGGCTCTGCTCTCTCT | GTTGGAGCTTTCCTCATCCA |
| RTDR1 | AGGCAGTGAGTGGTTTCTGC | CTCACAGAGGTGAATGAAGGG | GTCGCCTGGCAACTGAATAG | AAGAGACTTAGCACATTTATTCACTCA |
| SBF1 | CCGCGTCCCTCGCCAT | GGTAACGACCGGAAGCAGAG | GGCTGGCTGGGAAGATG | CTATTTACAGGCCCATTGCG |
| SCA10 | CTCCTCGCCTTCCTCCTC | TCCATGAAACAGATTCCAAAAA | CGGTTAGGGCTGTGTAGGG | CACTTGCAATTTCACATACGG |
| SCO2 | GATCCATGCTGCTGCTGAC | GCAGTGGCTCAAGACAGGAC | GCTTCCTCTCGTGCTTGGT | TAAACGCAGCCCGTTTAATG |
| SDF2L1 | GCCGGGGCGATGTGGAGC | AAACCCAAAGTCTCTGCCAA | TGGGCCCGAGGGGCTGGAGC | AGGCACTTGAGGACCCCTAC |
| SEC14L2 | GACGAGTCTGTGCTCCATCA | CAGTTTCTTTGGGCTTCAGG | AAGGCTGGGACTTTACTCCG | TCGCTCTTCCTCTGCCTAAC |
| SEP3 | CTGGAGGAACGGAGACAAAG | CACAGGCTCTCTCGCTCTCT | GAGCATCTCCCTGGAGGAAC | TTGGGACACAAGGAAAGGAG |
| SERPIND1 | TAATGCTGTGAGGGCCTCTT | CAAGCCTCTCTTCTTGGTCG | AGTCCCACATCAAAGGTTGG | ATGAGCTTACAGCATGGGCT |
| SEZ6L | CCCTGCAGCCACGATG | GGTTGAGTTCACAAGTCCCCT | CCCTTTCTCGCTCACCG | GAAACATCCAAACCCACACC |
| SF3A1 | TCTTGCGAGCTCGTCGTACT | AACAAATATGCAGGCAAGGC | CTTCAGCCTTCGTGTCTGGT | TCCCAAACTGAGTAAGAGCGA |
| SH3BP1 | GTGACCCCGCAGCCCC | AGAGGATTGTCCTGAGCCCT | AGGAGAGGCAGGCTGGAC | GAATCAGTCCCGTAGGAGCA |
| SLC16A8 | GGAGAAGGAGACTTGGGAGG | ATAGCAGCTTCACTGGCGAT | AGAGGTGCAGAGTCAGGTGG | TCACCAGTTTCCTGTTGCTC |
| SLC25A1 | TCTCGGACCCGAAGCC | GTCACACACAGACCACAGGG | GAGCGCGGAGTTCTGGAG | CACAGGGTCATAGGCCAGAT |
| SLC25A17 | TTTCCTAACTCCACTGGCTG | CATACTCCCTGTGCACCCTT | GATTGCGACTCTCACACCCT | CATTAGTCCAACTGCCACCC |
| SLC25A18 | GCTCTGTCCACACTGCTACG | CACTACAACGCAGAGGACCA | AGATGAACGTCGACTCGCTT | TGTTTCTCCCGAGATTGAGG |
| SLC5A1 | GTATAAGGAGCTAGCGGCCC | TTCAACACCACAGGACGAGA | CTCCCTCAAAGTCCCAGGTC | TGAGTGGACTTCCCTTCTGC |
| SLC5A4 | CAGCCATGGCCAGTACGGTTAGCC | GGCTCAGATAGAGTTCAGGCA | CTGCCTGCAGCCATGGCCAG | GAATTATTCATTATTCTAATGGCTCAG |
| SLC7A4 | GTAGCTGGCTCGGTGCTCT | CCCCTGGATGAAGGTCCTAA | CAGCAGCAGGTTCCAGTAGC | GGCTGGCCAAGCAATTATTA |
| SMARCB1 | CAGCCCTCCTGATCCCTC | CAACAAATGGAATGTGTGCC | ATTTCGCCTTCCGGCTTC | CTGTTACTAGCCCTGCCTGG |
| SMC1L2 | GGTGAGGCGTGGAGGG | CAGCCTCTTTGGCTAGAACG | CTTGATAACGCGGGTGAGG | TGCAAGGCAAGATCAGAGTG |
| SMTN | CTCACCAGAAAGGAACCGAC | AGGGTGTCGCAACAGACAG | TCTAATCCGTCTGTCGGGTC | AAACGCTGCGTGTGTATGTG |
| SNAP29 | TCTGTTTCCCAGACCGAGAG | GCTGGAAGAACTTGGTGAGG | GCTCCTCCTTCTGTTTCCC | ATCAAACGCCCAAGATATGC |
| SNRPD3 | TGGAATTCTGGGTGTTAGGC | AAGCACCCACTCCAATGAAC | GAGTGAGGAGGAAGCGGAG | GCCAAAGCAAACTTCAAATG |
| SOX10 | GTTGGACTCTTTGCGAGGAC | GACCTGTCAGCCTCTTCAGC | CACTTCCTAAGGACGAGCCC | CTCTGTCCAGCCTGTTCTCC |
| SREBF2 | GTGTCATGGGCGGTGG | AGAAGACAAGGCTCTTCCCC | GTTGTCGGGTGTCATGGG | TGGGCATCTAGTGACAGCAG |
| SSTR3 | AAGAAAAGACGGCACCTCAA | AAGTAGGCCCTAGGCACACC | AGATGGGCAAATGGAGAATG | TACCCAAGGTCACACAGCAA |
| ST13 | TCTAGTCTGTTCTGTCTTGCGG | TTCCTTCAGCAAGGGCTTTA | CGCAGAGGGAGTAGGAATGA | TTGCGACATCCATAAGGTGA |
| STK22A | GTCGCTCCTGGCACCAT | TGTCATGCTAGGTGCTTGCT | GACGCCTCCGGTAGTGTAAA | ACTTGCACCTGTGCCTTCTT |
| SULT4A1 | CATGGCGGAGAGCGAGG | GACTGTCTGGGTATTGTGAGC | GGCTGCGAGCCGGG | CTCCCTCCGCTCACGC |
| SYN3 | TACCACTCCCTGTACCTGGC | CAGGAACCAAGGCTGAGAAG | CGCAGGTAAATAAAGGCAGC | GTATGTTCTCAGGCCCTCCA |
| SYNGR1 | GTGCAGCCACGATGGAAG | GTGTGGAGAGAGGGGACAGA | GGGTGCAGCCACGATG | TATGACCAAGCCACGGTGTA |
| TAB1 | AGATGGCGGCGCAGAG | GTTAGGAAAAGGCTGGACCC | GGTTCCTCCAAGATGGCG | AACACTCAGGGTTCACAGGG |
| TBX1 | AGGGCTCAGGGTCCTCC | GGGAAACACGACAACTCCAT | GAGCGAGGAGGAAGGGAAC | TGGAAATTTCAAACATGAACCA |
| TCF20 | TTCTAGGAGGGCTGTTGGC | ACCTGTGCTTGCTGTCCTTT | TTGTACCAAGGAGATGCGTG | CATTCCAACGTCTTGGGTCT |
| TCN2 | TTTCCCGATTCTTGCTCACT | GGAAGATGCTTGGCTCTCTG | ACCAGCTGTGGTCAGGAGAG | CCAGACTTGGCCAGAAAGAC |
| TEF | GAGTCCGGGGCACGAT | GCAGAGGTCTGAGGTGCAG | GGCTCCGGCCCATCTC | CCCTTCTGATTCAACTGGGA |
| TIMP3 | ACTTTGGAGAGGCGAGCAG | GCCAGGAGGATAGTTCCCA | CTCTTGCTCCTCCAGCTCCT | CCCAGGTGGAAATGAATGAT |
| TOB2 | GAAGGAAAGGAGAGCGACCT | TTTCTGTGGTCTTGGGTGCT | CCGACCTCCTAAGAGCTGAA | ACACTTGGGTGCTTTGCAG |
| TOM1L1 | GTGGCAGCGGCGGTAG | AGCCTAAGGGTGTGAGAGCA | GTTGCTGTCAGCTGATTCCC | GCCTCCACTTCTCAGCAAAG |
| TOP3B | AACTGGAGTGTGAAGGACCG | TTGAAGTGCCATGAGGTGTC | TCTTGCCTCAGGTTGGTCTT | TTTAATAGTTTGAAGTGCCATGAG |
| TPST2 | AAGGGAGATGTGAGCCTGG | AGTCATTTGCGGAGATCTGG | GGAGCCCAGAGATGAGAGTG | TGGCGATTAATAGCAAGCAA |
| TR | GCAGCCCTAGCTGCCC | ACGCTGACCATCTCACAGG | CCACACCCTATCCCAGTGTT | CAGAGGCTGAGAGGTGCAG |
| TST | CACACACCGACTTCCTTCCT | CAGAGAGAGGGTGAGCCTTG | TCCAGAGGGAATCTGCAAAC | GGCAAAGTTTATTCCAGTGTTG |
| TTLL1 | GATTATGGCAGGGAAAGTAAAATGGG | CAGGGCTTAGGAAAGGAAAAAAGC | CAGTGACGTCAGCGAGACC | CCAAAGAAGTGCCTTCG |
| TUBA8 | CTGTATCTGGAGCAGTCGGG | GCTTTGAATGGCACAGCATA | GTGTGTCACAACGGCGGA | CTGGGTAAGGCAGGAATCAG |
| TXN2 | CTGTACCCGGAAGTGACGTT | AAGGGAACCAGGACTCATCC | GAGAGGGACGCTCTTCCG | CATAGGCCCTAGAAGGAGGG |
| UBE2L3 | AAGGAGCAGCACCAAATCC | TCTCTGCTCACACTTGCTGG | GGGAAGGAGCAGCACCAA | GCAAGGGTAGGGAAGAAAGG |
| UFD1L | AGGTGGTGTCCATCATGTTCT | CAATCAGCCAACAGTCCTCA | GTTTCTTCGTTGCATTGCCT | TTTCCAATCAGCCAACAGT |
| UPK3 | GGGCGATGCCTCCGCTCT | AAGTCAGGGTGTGACAACCAC | CCGCGCTCTGGCGGCTCCTC | AAGATTTTATTAAGGGTTTTCCTGC |
| VPREB1 | CATGTCTGCACCATGTCC | ATGACAGTCTCTCCAAGGGG | AGAGCTCTGCATGTCTGCAC | CCACCCTCTTCCATGACAGT |
| VPREB3 | TCTACTTGCCTGCCTCCCT | CAAGGTCAGGGGCAGAAAT | CCTGGAGCATATAGCCTTGC | GGAAGCAAGGCTCAGAGAAA |
| XBP1 | GCTATGGTGGTGGTGGCAGC | CCACATTAGCTTGGCTCTCTG | TCTGGAGCTATGGTGGTGGT | TGAGAGGTGCTTCCTCGATT |
| YWHAH | AGCCAGCGGTGTGAGG | ATTGTGGCAAGGAAGAATCG | GTCTCCTCCCTCGGCGTT | CTGAGTAGCTGTGCTGCCAA |
| ZNF278 | GAGTGTGGACACGTCTGCTG | TGGAGAAGGAGTTGGAGTGG | TAGACAGTCTGATCCGGGCT | AGGAATTCCAGCTTCTTCCC |
| ZNF279 | AGGAAGCATTGGGACTTGTG | CTGAAACTAGAATTAATGGGACT | AAACCTGAGGCTGGGAAGTT | TTGAAATACTTGCTTTAGATTTACTGAA |
| ZNF280 | TGCACTCGCAACTTTAGGAA | TCACTTCTGCCTTTCGGAAC | GCTGCACTGAATCATCTGGA | ATGGCCTAGCCTCACTTCTG |
| ZNF70 | TCTCTGGAGGATGGGATGAC | TTGTGTGGGCTCCTCTTTCT | CTAAGAGTTTGCAGGGTGCC | GACGTGGAATAAAGGCTCCA |
| ZNF74 | GATCCTGGAGGCTACACAGC | AACTAGAGGAGGCACCCTGG | GCAGTACTCGCTCTTCAGGG | AACAACTTCCAGGCCCATC |
