# Supplementary material for: A genome annotation-driven approach to cloning the human ORFeome
Source: Genome Biol. 2004 Sep 30;5(10):R84. doi: 10.1186/gb-2004-5-10-r84 (PMC545604; doi:10.1186/gb-2004-5-10-r84)
Supplement: Additional data file 5 — The results of nonparametric ANOVA (Kruskal-Wallis Test) for chromosome 22 genes isolated as cDNA by the method described here only (SANGER), found in the cDNA collections only (OTHER), isolated by both ourselves and the cDNA collections (BOTH) or not isolated (NOT) [file gb-2004-5-10-r84-s5.doc]

| **Class Comparison** | **GC** | | **Length** | | **Expression Diversity** | |
| --- | --- | --- | --- | --- | --- | --- |
| BOTH vs SANGER  BOTH vs OTHER  BOTH vs NOT  SANGER vs OTHER  SANGER vs NOT  OTHER vs NOT | Mean Rank Difference | P value | Mean Rank Difference | P value | Mean Rank Difference | P value |
|  | -6.512  -100.67    -59.410  -94.156  -52.899  41.257 | P>0.05    P<0.01    P<0.01  P<0.01  P<0.05  P>0.05 | -21.163  -60.886  -105.10  -39.723  -83.939  -44.216 | P>0.05  P>0.05  P<0.001    P>0.05    P<0.001    P>0.05 | 25.390  -14.815  18.063  -40.205  -7.327  32.878 | P<0.05    P>0.05  P>0.05    P>0.05  P>0.05  P>0.05 |

Results of non parametric ANOVA (Kruskal-Wallis Test) for chromosome 22 genes isolated as cDNA by the method described here only

(SANGER), found in the cDNA collections only (OTHER), isolated by both ourselves and the cDNA collections (BOTH) or not isolated (NOT).

Mean Rank differences and P values are given after Dunn's multiple comparisons test. Significant values referred to in the text are coloured red.
